# CRISPR/Cas9 and Transgene Verification of Gene Involvement in Unfolded Protein Response and Recombinant Protein Production in Barley Grain

**DOI:** 10.3389/fpls.2021.755788

**Published:** 2021-11-15

**Authors:** Michael Panting, Inger Baeksted Holme, Jón Már Björnsson, Yingxin Zhong, Henrik Brinch-Pedersen

**Affiliations:** ^1^Department of Agroecology, Research Center Flakkebjerg, Aarhus University, Slagelse, Denmark; ^2^ORF Genetics, Kópavogur, Iceland; ^3^National Technique Innovation Center for Regional Wheat Production, Key Laboratory of Crop Physiology and Ecology in Southern China, Ministry of Agriculture, National Engineering and Technology Center for Information Agriculture, Nanjing Agricultural University, Nanjing, China

**Keywords:** barley, unfolded protein response, heterologous expression, recombinant protein, CRISPR

## Abstract

The use of plants as heterologous hosts to produce recombinant proteins has some intriguing advantages. There is, however, the potential of overloading the endoplasmic reticulum (ER) capacity when producing recombinant proteins in the seeds. This leads to an ER-stress condition and accumulating of unfolded proteins. The unfolded protein response (UPR) is activated to alleviate the ER-stress. With the aim to increase the yield of human epidermal growth factor (EGF) and mouse leukemia inhibitory factor (mLIF) in barley, we selected genes reported to have increased expression during ER-induced stress. The selected genes were calreticulin (CRT), protein disulfide isomerase (PDI), isopentenyl diphosphate isomerase (IPI), glutathione-s-transferase (GST), HSP70, HSP26, and HSP16.9. These were knocked out using CRISPR/Cas9 or overexpressed by conventional transgenesis. The generated homozygous barley lines were crossed with barley plants expressing EGF or mLIF and the offspring plants analyzed for EGF and mLIF protein accumulation in the mature grain. All manipulated genes had an impact on the expression of UPR genes when plantlets were subjected to tunicamycin (TN). The PDI knockout plant showed decreased protein body formation, with protein evenly distributed in the cells of the endosperm. The two genes, GST and IPI, were found to have a positive effect on recombinant protein production. mLIF expression was increased in a F_2_ homozygous GST knockout mutant background as compared to a F_2_ GST wild-type offspring. The overexpression of IPI in a F_1_ cross showed a significant increase in EGF expression. We demonstrate that manipulation of UPR related genes can have a positive effect on recombinant protein accumulation.

## Introduction

The production of recombinant proteins in plants are of growing interest, but the yield from plants is mostly not competitive to traditional host systems like bacteria, yeast, and CHO cells. Plants optimized for recombinant protein production are therefore in demand. Improvements for plant expression is mainly focusing on external factors like promoters, codon optimizations, and on engineering the glycosylation pattern to mimic mammals (Strasser et al., 2008; Habibi et al., 2017). Fewer efforts have focused on improving the tolerance of the plant host organism for heterologous protein production, a shortcoming compared to bacteria and yeast, where different strains have been engineered to improve yield (Inan et al., 2006; Zhang et al., 2006; Chou, 2007; Damasceno et al., 2007).

Already established barley lines producing either a human epidermal growth factor (EGF) or murine leukemia inhibitory factor (mLIF) in the seeds were used as a starting point for this study. EGF is a 53 residue peptide with six cysteine residues forming three intramolecular disulfide bonds (Harris et al., 2003). EGF is used in cell cultures for proliferation and as a medical agent to heal cut and burn wounds (Andree et al., 1994). Different EGF expression hosts have been used previously, with tobacco leaves being the most common for plants (Wirth et al., 2004; Bai et al., 2007; Torrent et al., 2009). mLIF is another high-value recombinant protein. It is used in stem cell research to maintain and proliferate embryonic stem cells and induced pluripotent stem cells. It accounts for up to 90% of stem cell propagation costs (Youngblood et al., 2014). LIF is a 180 amino acid ligand, binding to the LIF receptor and the gp130 receptor forming a heterodimeric complex activating the JAK/STAT pathways and the MAPK cascade in the cytosol of the cells (Heinrich et al., 2003). Commercial LIF recombinant protein is traditionally expressed in *Escherichia coli* (*E. coli*), but there is also recombinant human LIF (hLIF) on the market produced in rice (Merck), barley (ORF Genetics), and recombinant mouse LIF (mLIF) produced in barley (ORF Genetics). The hLIF from rice has similar biochemical properties as LIF derived from *E. coli*, but with a significant lower endotoxin level (Youngblood et al., 2014). Similarly, the bioactivity of mLIF and hLIF from barley is fully comparable with corresponding LIF proteins on the market and have low endotoxin levels.

The production of recombinant proteins passing through the endoplasmic reticulum (ER) can lead to an overload of the protein maturation processes, resulting in accumulation of unfolded and misfolded proteins. This increase in unfolded protein leads to initiation of the so-called unfolded protein response (UPR), helping to alleviate ER-stress by upregulating the expression of chaperone genes and genes involved in ER-associated degradation (ERAD). The UPR also downregulates the synthesis of secretion proteins to reduce the load of misfolded proteins (Thomas and Walmsley, 2015; Wan and Jiang, 2016).

To improve barley as a host organism, we studied candidate genes based on a proteome study of the mechanisms of protein secretion using barley aleurone layer as a stress model platform (Barba-Espin et al., 2014). The main criteria for selection of candidate genes were a clear induction of protein synthesis during tunicamycin (TN)-induced stress. TN is a known ER-stress inducer acting by blocking the initial step of glycoprotein synthesis through inhibition of GlcNAc phosphotransferase. The seven candidate genes selected for this study had an increased translation when aleurone layers were incubated with Giberillic acid (GA_3_) and TN (Barba-Espin et al., 2014). These were Protein disulfide isomerase (PDI), Calreticulin (CRT), heat shock proteins (Hsps) Hsp70, Hsp16.9 kDA, Hsp26kDa, Glutathione-S-transferase (GST), and Isopentenyl diphosphate delta isomerase (IPI). The first five genes have also previously been described as involved in processing of proteins, i.e., chaperones such as PDI, CRT, and HSPs. Two of the candidate genes (HSP16.9 kDA, HSP26 kDa) are small heat shock protein (sHSP) chaperones that bind non-native proteins in order to prevent aggregation. By binding, the sHSP keeps the misfolded protein in a foldable state for other proteins such as HSP70 to bind and refold them (Montfort et al., 2001). In plants, the sHSPs is a multigene family with five classes localizing to all major compartments of the cell (Waters and Vierling, 1999). The class of HSP16.9 is identified as belonging to the cytosolic class I, while the class of HSP26 is most likely also present in the cytosol because it has no signal peptide. All sHSPs share a central α-crystallin domain of 80–100 residues, with the N and C-terminal extensions being highly variable and gives the different sHSPs their substrate specificity and facilitates oligomerization (de Jong et al., 1998). A *HSP70* gene was also selected as a candidate because these are known to function during ER-stress in plants (Noh et al., 2003). HSP70 functions by binding nascent unfolded or misfolded proteins and refolding them into a native state in an ATP-dependent manner. It also interacts with other chaperones such as sHSPs and the HSP70 protein binding immunoglobulin protein (BiP) binds to CRT in the ER (Crofts et al., 1998). PDI catalyzes the formation of disulfide bonds between cysteine residues or reshuffles them if they are not correctly paired. The expression of PDI is known to be induced during UPR to help with proper folding of unfolded or misfolded proteins (Tu and Weissman, 2004). CRT is part of the calreticulin-calnexin cycle, helping with Ca^2+^ homeostasis, N-glycosylation of glycoproteins through oligosaccharide modifications, and as a quality control system. The obtained CRT sequence from the initial study aligns with the partial CDS sequences *CRT1* and *CRT2* (L27349.1 and L27348.1) from barley, but it only aligns to one gene in the genome (HORVU2Hr1G121990). This has 70.75% ID to the *Arabidopsis AtCRT2* and 70.59% ID to *AtCRT1*. Glutathione (GSH) is a low weight antioxidant, functioning as a redox buffer throughout the cell. GST reduces organic peroxides by conjugating of GSH, helping to mitigate oxidative stress. The Lambda and DHAR GST groups function as dethiolating proteins, removing S-glutathione from bound proteins in a mixed disulfide bond formation (Dixon et al., 2002; Uzilday et al., 2018). Based on sequence alignment, the GST selected here has the protein ID ALH06514.1 which includes the C-terminal alpha helical domain of the GST Lambda class (cd03203). It also aligns to the *AtGST* Lamda class genes in *Arabidopsis thaliana*. The last candidate included is IPI, an enzyme that is part of the mevalonate pathway and needed for synthesis of the polyisoprenoid lipid carrier of glycans for N-glycosylation (Jones et al., 2009).

The current study had two prime objectives. First we wanted to study barley candidate genes for their involvement in UPR through inducible ER-stress. This was done with an assay on germinating seeds, introducing TN during germination to induce ER-stress and initiate the UPR, mimmicking the high protein synthesis situation of grain maturation. Secondly, we wanted to increase the yield of recombinant proteins in the barley grain by improving the hosts capacity to handle increased expression of a recombinant protein. To achieve this, we crossed knockout and overexpressing lines of the UPR-affected candidate genes with already established barley lines producing either human EGF or mLIF. The hypothesis behind was that genes with a positive effect in alleviating ER-stress will also have a positive effect on recombinant protein synthesis while they are passing through the ER during grain development. The two recombinant proteins were selected due to their differences in post translational modifications (PTMs). EGF is a small peptide with three disulfide bonds and no glycosylation, while mLIF also has three disuplhide bonds, but hosts several predicted glycosylation sites.

## Materials and Methods

### Vector Constructions

Protospacers for the synthetic guide RNA (sgRNA) in the CRISPR/Cas9 experiments were designed to target each of the seven candidate genes (Supplementary Table 1). For screening purposes, protospacers with a restriction enzyme recognition site spanning the fourth base from the protospacer adjacent motif (PAM) were prioritized. A list of primers used to amplify the targeted region can be seen in Supplementary Table 2. Amplification was done using the Phusion^TM^ High-Fidelity DNA Polymerase (Thermo Scientific) as described by the manufacturer.

The protospacer sequences were cloned into the entry vector pJG85 under the control of the wheat U6 RNA polymerase III promoter (TaU6), flanked by the attL5 and attL2 Gateway cloning sites (Gil-Humanes et al., 2017). Another entry vector pJG80 (Gil-Humanes et al., 2017), with a promotor-less wheat optimized Cas9 (TaCas9) flanked by a attL1 and attR5 Gateway cloning site, was cloned, along with pJG85 using Gateway LR clonase enzyme mix (Invitrogen) into the destination vector pANIC6A described by Mann et al. (2012) (Supplementary Figure 1A).

For overexpression of candidate genes, the WBVec8 modified vector Ubi:USER:NOS was used with a maize ubiquitin constitutive promoter (Hebelstrup et al., 2010) (Supplementary Figure 1B). The vector was linearized using *Pac*I leaving a 3′-AT overhang. The genes were either amplified from cDNA or genomic DNA with primers that had a 15 nt overhang homologous to the vector insertion site and AT overhang nucleotides (CAGGCTGAGGTCTTAAT) (Supplementary Table 3). The Phusion^TM^ High-Fidelity DNA Polymerase (Thermo Scientific) was used for amplification as instructed by the manufacturer. The fragments were cloned into the vector using In-Fusion^®^ HD kit (Clontech) as described by the manufacturer. The gene sequences amplified for overexpression were obtained from either genomic DNA or cDNA of barley cultivar Golden Promise (GP). The sequences are listed in the Supplementary Data 1 with the primers used for amplification marked on the sequences. The annotations used from either the old or new genome assembly are stated.

The vectors were cloned into Stellar^TM^ competent cells (Clontech) using heat shock transformation. Colonies were grown at 37°C on selective LB medium with 50 μg/ml spectinomycin or kanamycin for overexpression or CRISPR/Cas9 constructs, respectively.

All vectors were subsequently transformed into *Agrobacterium* strain AGL0 using the freeze/thaw method. Colonies were grown at 28°C on selective medium with rifampicin (25 μg/ml) and 50 μg/ml spectinomycin or kanamycin for overexpression or CRISPR/Cas9 constructs, respectively.

#### Transformation

Golden Promise plants were grown in growth chamber with a 16 h light period with 350 μ E m^–2^ s^–1^ and 15 and 10°C day and night temperature, respectively. Twelve- to fourteen-day-old embryos were isolated and transformed by *Agrobacterium*-mediated transformation using the procedure described in Holme et al. (2012).

### Molecular Analysis of Transformants

Crude DNA extraction from a small piece of leaf was performed using the Extract-N-Amp^TM^ Plant PCR kit (Sigma Aldrich), as described by the manufacturer. The crude DNA was diluted 1:3 before used as template for the polymerase from the same kit or the KAPA3G Plant PCR kit (Kapa biosystems).

DNA for cloning and sequencing was extracted from 10 cm young leaf pieces immediately frozen in liquid nitrogen. The leaves were crushed in a FastPrep-24 5G homogenizer (MP Biomedicals) while still frozen and DNA extracted by the phenol/chloroform extraction method.

Plants obtained by CRISPR/Cas9 transformation and by overexpression of the candidate genes were screened by PCR amplification of the hygromycin resistance gene in the T-DNA. The same primers were used for amplification of hygromycin in the knockout and overexpressing transformants Hyg_fw: 5′-ACTCACCGCGACGTCTGTCG-3′ and Hyg_rv: 5′-GCGCGTCTGCTGCTCCATA-3′. The program was 95°C for 3 min, then 40 cycles with 95°C for 3 min, 61°C for 15 s, 72°C for 40 s, and a final extension at 72°C for 1 min.

The product sizes were different in the knockout and overexpression constructs due to an intron in the hygromycin gene in the overexpression vector. The PCR products were 727 bp for knockout mutants and 917 bp for overexpression transformants.

Transformed plants obtained by CRISPR/Cas9 transformation were analyzed for the presence of mutations by PCR/restriction enzyme (PCR/RE) analysis as an initial screen to identify mutations. Uncut PCR products from the PCR/RE analysis of the CRISPR/Cas9 transformed plants were cloned and sequenced. Cloning was done using the Zero Blunt^TM^ TOPO^TM^ PCR cloning kit (Invitrogen). In the T_1_ generation, progenies homozygous for the knockout mutations and no T-DNA insert were identified and selected for further analysis and for crossing.

### Crossings

The barley lines expressing EGF and mLIF were kindly provided by ORF genetics (EGF*^*ORF*^* and mLIF*^*ORF*^*, respectively). They were generated by *Agrobacterium*-meditated transformation of Golden Promise. The T-DNA within the EGF*^*ORF*^* line contained two human EGF copies driven by a seed-specific B-hordein promoter or an oat globulin seed-specific promoter, respectively. Both copies were terminated with a D-hordein terminator (Supplementary Figure 1C). The T-DNA within the mLIF*^*ORF*^* line contained only a single copy of mLIF controlled by a seed specific D-hordein promoter and terminator (Supplementary Figure 1D). The EGF^*ORF*^ line was homozygous after double haploid formation whereas the mLIF*^*ORF*^* was heterozygous.

#### Crossing Conditions

Plants used for crossing were grown in pots in an open-air semi field growth setup supplied with water and nutrients. The EGF*^*ORF*^* and mLIF*^*ORF*^* barley plants were emasculated and pollen from knockout or overexpression plants were manually transferred to the emasculated plants after 3–4 days. The progeny was screened using PCR, PCR/RE, and/or sequencing to identify positive crosses.

#### PCR Screening of Crosses

Differences in the sequence of the hygromycin resistance gene used in the EGF*^*ORF*^* and mLIF*^*ORF*^* plants and the knockout and the overexpressing plants allowed for the design of specific primers for amplifying the T-DNA in EGF*^*ORF*^* and mLIF*^*ORF*^* plants (HPT_fw 5′-CCGACCTCATGCAACTCT-3′ and HPT_rv 5′-CTTCTCACTCCTTGGCCCT-3′). The program was 95°C for 3 min, then 40 cycles with 95°C for 3 min, 62°C for 15 s, 72°C for 40 s, and a final extension at 72°C for 1 min on a crude DNA extract. The amplified product was 1,154 bp.

### Grain Analysis

Grain from transformed lines were counted and weighed for calculation of the 1,000-grain weight. Five g of grains from some of the lines were also sent to Eurofins for determination of total protein content.^1^ This was done using the Dumas combustion method to determine the total nitrogen content in the samples.

### Endoplasmic Reticulum-Stress Assay

The ER-stress assay was modified from a method described for *Arabidopsis* (McCormack et al., 2015). Two barley seeds per well in a 12-well plate were germinated in 800 μl 1/2 MS liquid media with 50 μg/ml ampicillin and incubated at 23°C, with 16/8 light/dark hours. After 4 days, the media was removed and 1.6 ml fresh media with either 0, 25, or 100 μg/ml TN dissolved in dimethyl sulfoxide (DMSO) was added. The plantlets were incubated for another 4 days, after which the media was removed. The wells were washed three times in sterile water before 1.6 ml media without TN was added to each well. After three more days of incubation, the plantlets were transferred to 2 ml Eppendorf tubes, frozen in liquid nitrogen and stored at −80°C until RNA extraction.

### RNA Extractions

Frozen leaves were crushed using two glass beads in a FastPrep-24^TM^ 5G homogenizer (MP Biomedicals) for 6 s at speed 5.0. RNA extraction was done using the Spectrum^TM^ Plant Total RNA Kit (Sigma Aldrich) as described by the manufacturer. Potential residual DNA was removed by addition of RNase free DNase from Qiagen along with RNasin^®^ Ribonuclease inhibitor (Promega). Subsequently, the samples were purified using the NucleoSpin^®^ Gel and PCR Clean-up kit (Macherey-Nagel) with the NTC buffer. RNA concentration and quality were evaluated by NanoDrop 1000 (ThermoFisher Scientific) and on a 1% agarose gel to visualize the 18S and 28S ribosomal bands.

#### First Strand cDNA Synthesis

The first strand cDNA synthesis was done using the SuperScript^®^ IV Reverse Transcriptase kit from Invitrogen with one μg total RNA as template. RNasin^®^ Ribonuclease inhibitors (Promega) were used as RNase inhibitor, following the instructions of the manufacturer. A 9N random primer was used for amplification for the ER-stress assay and overexpressing transformant screening and a 4:1 9N random primer and oligodT primer mix was used for generating the cDNA template used for the overexpression CDS constructs. The program for synthesis was 10 min at 23°C, 20 min at 50°C, and 10 min at 80°C.

#### Expression Analysis by RT-qPCR

RT-qPCR of target genes in the ER-stress assay was performed using a ViiA 7 Real-Time PCR System (Applied Biosystems). Experimental setup and data extraction was done using the QuantStudio^TM^ Real-Time PCR Software v. 1.3. The expression of the UPR genes *PDI*, *CRT*, and *BiP* were determined for all knockout mutants and overexpression transformants. Each sample was analyzed in both biological and technical triplicates for nine data points per barley line using the program suggested by the software. Samples with insufficient amplification or multiple melting curve peaks were omitted for further analysis. An RNA triplicate of all samples was analyzed with the actin reference gene as a control for DNA contamination.

A similar procedure was performed for the RT-qPCR of T_0_ and T_1_ overexpressing transformants, except for the T_0_ plants that were only analyzed in a technical triplicate.

The RT-qPCR amplifications were performed using Power SYBR^TM^ Green PCR Master Mix (Applied Biosystems). For each sample, 6 μl SYBR Green Master mix, 2.4 μl Primer mix (1.5 μM each), 2.6 μl water, and 1 μl cDNA were mixed to a total volume of 12 μl.

The expression of each target gene (ΔC_T_) was calculated by subtracting the mean C_T_ value of the reference gene(s) from the C_T_ value of the target genes. For screening of the overexpressing transformants, the −ΔC_T_ was calculated by subtracting the reference gene *SP2* from the gene of interest. In the ER-stress assay, the expression of the investigated UPR genes *PDI*, *CRT*, and *BiP* were normalized to the reference genes *actin* and *GAPDH* (ΔC_T_). The expression was determined relative to the expression in GP (-ΔΔC_T_) at each TN concentration. The standard error was used as error bars and a two-tailed Student’s *t*-test on the mean −ΔΔC_T_ was done to find significant differences between the GP control and the knockout mutant and overexpression lines. *P* < 0.05 was regarded as significant.

Primers used for RT-qPCR are listed in Supplementary Table 4. Primer set for the *actin* reference gene (qPCR_actin), was described in Kaczmarczyk et al. (2012).

### Sandwich ELISA Assay for Recombinant Protein Content

The extraction and analysis of recombinant EGF protein was performed as described in Panting et al. (2020). The Sandwhich ELISA assay of mLIF was done with a mouse LIF DuoSet ELISA kit from R&D Systems as described by the manufacturer. The samples were diluted 32,000 times before measuring the amount of mLIF protein. The mean μg/ml concentration of each biological replicate was calculated, and the standard error and a two-tailed Student’s *t*-test was calculated based on the mean concentrations. *p* < 0.05 was considered a significant change.

### Sectioning for Microscopy

Knockout PDI mutant plants and GP controls were grown in the semi field and harvested at 20 days after pollination (DAP) for protein staining of the starchy endosperm. The method used for staining protein and sectioning was basically as described in a previous study (Wan et al., 2014). Samples were gradually dehydrated in ethanol dilution series going from 10 to 100% ethanol, increasing by 10% at each step with incubation for 1 h. Samples were kept overnight in 70% ethanol and dried two times in 100% ethanol. Sections were infiltrated with LR White resin using a mild vacuum for 1 h. The ethanol:LR White resin ratio went from 4:1 to 1:4 and the samples were kept at each concentration over night at 4°C before entering the next step. Finally, the samples were infiltrated with pure LR White resin. The pure LR White resin step was repeated three times and changed up to two times per day for 4 days or longer. Samples were incubated at room temperature on a rotator during the day and kept at 4°C at night. The embedded samples were polymerized at 55°C and cut into 1 μm slices with an ultramicrotome (Ultracut R, Leica, Germany). The sections were stained using 1% Napthol Blue Black in 7% acetic acid and visualized under a light microscope. Then, the stained sections were rinsed, dried, and photographed using a light microscope (DMLS, Leica, Germany) for protein body imaging. The experiment was done in triplicate.

## Results

### Stress Gene Knockouts

Regenerated CRISPR/Cas9 transformed T_0_ plants were screened for the presence of the T-DNA. Between 12 and 39 transformants were screened for the hygromycin gene and positive plants were screened for mutations using PCR/RE or directly sequenced. Plants with partial or no digestion of the PCR-product were selected for further characterization and the PCR-product from 5 to 13 plants were cloned and sequenced to identify the mutation (Table 1). The subsequent T_1_ plants from selected mutants were again screened by PCR/RE and for the presence of the hygromycin resistance gene in order to select T_1_-plants homozygous for the mutation but without the CRISPR/Cas9 T-DNA construct. The PCR-product containing the mutation from these T_1_-plants was sequenced to verify the heredity of the mutation (Table 1). The knockout lines were named with GP followed by the knockout gene in superscript small letters (GP*^*hsp*70^*, GP*^*hsp*26^*, GP*^*hsp*^^16.9^*, GP*^*gst*^*, GP*^*crt*^*, GP*^*ipi*^*, and GP*^*pdi*^*). Supplementary Table 5 lists all generated plants for this study, and which experiments they are included in. For the GP*^*crt*^* mutant line, a homozygous mutant was first identified in the T_2_ generation and GP*^*hsp*26^* was first identified in the T_1_ generation. Six out of the seven mutants selected for further study had a +1 nt insert. The last mutant line was a −1 nt deletion of *HSP16.9* in GP*^*hsp*^^16.9^*.

**TABLE 1:** Selected knockout mutants obtained with CRISPR/Cas9.

**T_0_ – ID**	**Mutation**	**KO region sequence**	**Confidence**
GP*^*hsp70*^*	+1	GGCCTTCCTtCAGCACAACCATCAACAACGC	5 clones
GP*^*hsp16.9*^* – allele 1	−1	CCGGCGTTCTCTGGCAACAGC-AGACGGCC	4 clones
GP*^*hsp16.9*^* – allele 2	+1	CCGGCGTTCTCTGGCGACAGCGtAGACGGCC	
GP*^*hsp26*^*			
GP*^*gst*^* – allele 1	+1	CCCCCGGAaCCGTCGCCATGGCCGCCGCAGC	6 clones
GP*^*gst*^* – allele 2	+1	CCCCCGGAtCCGTCGCCATGGCCGCCGCAGC	
GP*^*crt*^*	+1	ACCCGATAgTCTGTGGGTACAGCACCAAGAA	1 clone
GP*^*pdi*^*	+1	CTCCACTGaGACCGTTTGAGTCCTTCAAATC	10 clones
GP*^*ipi*^*	+1	GCCCGTCGAaCCAGTTCACCCCTCTCGGTCG	3 clones

**T_1_ – ID**	**Mutation**	**sequence**	**Confidence**

GP*^*hsp70*^*	+1	GGCCTTCCTtCAGCACAACCATCAACAACGC	10 clones
GP*^*hsp16.9*^* – allele 1	−1	CCGGCGTTCTCTGGCAACAGC-AGACGGCC	fw + rv PCR
GP*^*hsp26*^*	+1	TCCCACCAgGGCACACACTGAAATTCAATTC	fw + rv PCR
GP*^*gst*^* – allele 2	+1	CCCCCGGAtCCGTCGCCATGGCCGCCGCAGC	11 clones
GP*^*crt*^*^†^	allele 1 (+1)	ACCCGATAgTCTGTGGGTACAGCACCAAGAA	5 clones
	allele 2 (wt)	ACCCGATATCTGTGGGTACAGCACCAAGAA	
GP*^*pdi*^*	+1	CTCCACTGaGACCGTTTGAGTCCTTCAAATC	RE digest
GP*^*ipi*^*	+1	GCCCGTCGAaCCAGTTCACCCCTCTCGGTCG	9 clones

*The mutation found in the T_0_ and T_1_ generations and the sequence around the mutations site [protospacer adjacent motif (PAM) site underlined]. The sequences were validated by sequencing of either TOPO clones of PCR products or PCR products directly.*

*^†^A homozygous allele 1 mutant was obtained in the T_2_ generation.*

### Stress Gene Overexpressing Plants

Despite several transformation attempts on 450 and 175 embryos with the overexpression *CRT* and *HSP16*.9 constructs, respectively, it was not possible to obtain any transformed plants. Plants transformed with the other five target genes were regenerated and screened by PCR and for overexpression of the inserted gene in T_0_ or T_1_ plants (Supplementary Figure 2). From expression analysis of 6 to 12 overexpressing plant lines per construct, a high expression line from each construct was selected and homozygous lines generated for further analysis. These were named with GP followed by superscript large letters (GP*^*IPI*^*, GP*^*HSP*26^*, GP*^*PDI*^*, GP*^*GST*^*, and GP*^*HSP*70^*).

### Endoplasmic Reticulum-Stress Assay

The stress responses were evaluated in barley plants where the seven candidate genes one by one were either knocked out or overexpressed, except for the overexpression of *CRT* and *HSP16.9*. Expression of the known UPR responsive genes *BiP*, *CRT*, and *PDI* were monitored in control plantlets (Lu and Christopher, 2008; Kim et al., 2013). These genes have previously been correlated to the UPR. Plantlets from knockout mutants and overexpressing lines exposed to either 0, 25, or 100 μg/ml TN. In the GP control, an expected gradual increase in *BiP* and *PDI* gene expression was observed as the TN concentration increased. For *CRT*, the expression level was similar between 0 and 25 μg/ml TN whereas increased expression was observed at 100 μg/ml TN (Supplementary Figure 3).

**FIGURE 1:**
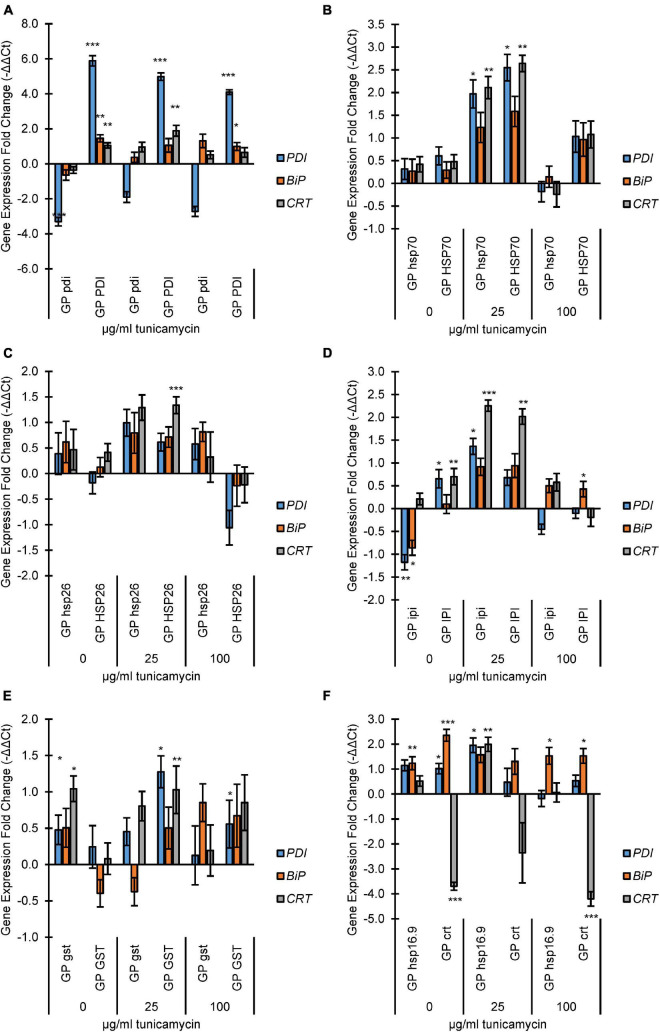
Endoplasmic Reticulum (ER)-stress analysis of mutant and overexpression lines by fold change expression of UPR genes protein disulfide isomerase (*PDI*), protein binding immunoglobulin protein (*BiP*), and calreticulin (*CRT*). The qRT-PCR samples were normalized to the expression level of two reference genes *actin* and *GAPDH*. The expression fold change levels were determined relative to Golden Promise expression at each TN concentration on a log_2_ scale (i.e., –ΔΔC_T_). Incubation concentrations of TN were 0, 25, or 100 μg/ml. **(A)** PDI lines; **(B)** HSP70 lines; **(C)** HSP26 lines; **(D)** IPI lines; **(E)** GST lines; **(F)** koHSP16.9 and koCRT lines. The error bars represent the standard error and Student’s *t*-test was applied to find any significant changes in expression between Golden Promise and mutant lines. *p < 0.05, **p < 0.01 and ***p < 0.001.

The PDI knockout and overexpressing lines GP*^*pdi*^* and GP*^*PDI*^* showed an expected lower and higher expressions of *PDI*, respectively, compared to GP (Figure 1A). Moreover, GP*^*PDI*^* had significantly increased expression of *BiP* at 0 and 100 μg/ml, and *CRT* expression was significantly higher at 0 and 25 μg/ml TN.

Both GP*^*hsp*70^* and GP*^*HSP*70^* showed an increased expression of all target genes at 25 μg/ml (Figure 1B). At 100 μg/ml TN, gene expression levels dropped again, with the lowest level seen in GP*^*hsp*70^.*

For the HSP26 knockout and overexpression lines, there was a general increase of expression at 25 μg/ml TN, but only *CRT* expression in GP*^*HSP*26^* was significant. At 100 μg/ml TN, stress gene expression levels in GP*^*hsp*26^* dropped to wild type levels whereas expression levels in GP*^*HSP*26^* were decreased to below wild type levels (Figure 1C).

In GP*^*ipi*^*, the *BiP* and *PDI* genes were upregulated at 0 μg/ml TN. Opposite, overexpression of *IPI* in GP*^*IPI*^* caused overexpression of PDI and CRT genes (Figure 1D). At 25 μg/ml TN, both GP*^*ipi*^* and GP*^*IPI*^* had increased expression of all genes, particularly, *CRT*.

In the *gst* knockout line GP*^*gst*^* the *BiP*, *CRT*, and *PDI* were increasingly expressed at 0 μg/ml TN (Figure 1E). The trend was less clear at 25 and 100 μg/ml although there were indications of increased *PDI* and *CRT* expression at 25 μg/ml and *BIP* at 100 μg/ml, respectively. For *GST* overexpressing plantlets, TN treatments at 25 and 100 μg/ml caused increased expression for the three studied genes, although only *PDI* and *CRT* at 25 μg/ml and *PDI* at 100 μg/ml had statistically significant.

As expected, the *crt* knockout in line GP*^*crt*^* led to no expression of *CRT* at any TN concentration (Figure 1F). In contrast, increased expression was seen for *BiP*, statically significant at 0 and 100 μg/ml TN. PDI expression in the *crt* knockout was only significantly increased in expression at 0 μg/ml TN. The GP*^*hsp*16.9^* mutant line had increased expression of *PDI* and *BiP* at 0 μg/ml TN. All three genes were increasingly expressed at 25 μg/ml and only *BiP* at 100 μg/ml TN.

In summary, the TN assay was functional in barley for evaluating the expression of the stress responsive genes *PDI*, *BPI*, and *CRT*. Treatments of wild type plantlets with increasing TN levels induced expression of the *PDI*, *BPI*, and *CRT* genes. Moreover, the stress response of the *PDI*, *BPI*, and *CRT* genes were modulated by individual overexpression or knockout of the seven genes *CRT*, *PDI*, *IPI*, *GST*, *HSP70*, *HSP26*, and *HSP16.9*. All lines showed at least one significant change in expression of at least one UPR stress gene, except for GP*^*hsp*26^*.

### Grain Weight

The effect of EGF and mLIF expression and stress gene overexpression or knockout on grain development was evaluated through 1,000-grain weight measurements (Table 2). First, expression of the two heterologous genes for EGF and mLIF caused no change in 1,000-grain weight. However, despite grown under the same growth conditions, only GP*^*ipi*^* showed the same 1,000-grain weight (45.9 g) as wild type (46 g). Grains from all other lines with modulated genes had a reduced 1,000-grain weight compared to GP. The lowest 1,000-grain weight was seen for GP*^*pdi*^* (29.5 g). Plants from the two overexpressing lines GP*^*HSP*26^* and GP*^*GST*^* grown in the greenhouse had a reduced 1,000-grain weight when compared to wild-type seeds grown under the same conditions. A general observation was that greenhouse conditions resulted in a lower 1,000-grain weight compared to the semi field.

**TABLE 2:** One thousand-grain weight of all transformants and controls.

**ID**	**Location**	**Grain counted**	**Weight (g)**	**1,000-grain weight (g)**	**Total protein (%)** ^†^
Golden Promise	Semifield	200	9.2	46.0	10.9
EGF*^*ORF*^*	Semifield	200	9.2	46.0	13.4
mLIF*^*ORF*^*	Semifield	200	9.3	46.5	
GP*^*pdi*^*	Semifield	200	5.9	29.5	14
GP*^*PDI*^*	Semifield	200	7.6	38.0	15.7
GP*^*hsp70*^*	Semifield	303	12.6	41.6	
GP*^*HSP70*^*	Semifield	310	11.1	35.8	
GP*^*hsp26*^*	Semifield	367	15.4	42.0	
GP*^*ipi*^*	Semifield	290	13.3	45.9	
GP*^*IPI*^*	Semifield	76	2.4	31.6	
GP*^*gst*^*	Semifield	226	9.9	43.8	
GP*^*hsp16.9*^*	Semifield	379	13	34.3	
GP*^*crt*^*	Semifield	268	10.7	39.9	
Golden Promise	Greenhouse	121	4.6	38.0	
GP*^*HSP26*^*	Greenhouse	90	3.1	34.4	
GP*^*GST*^*	Greenhouse	200	6.2	31.0	

*Location of growth is stated, and the total protein content of selected lines is presented.*

*^†^Determined using the Dumas method on 5 g of grain.*

**FIGURE 2:**
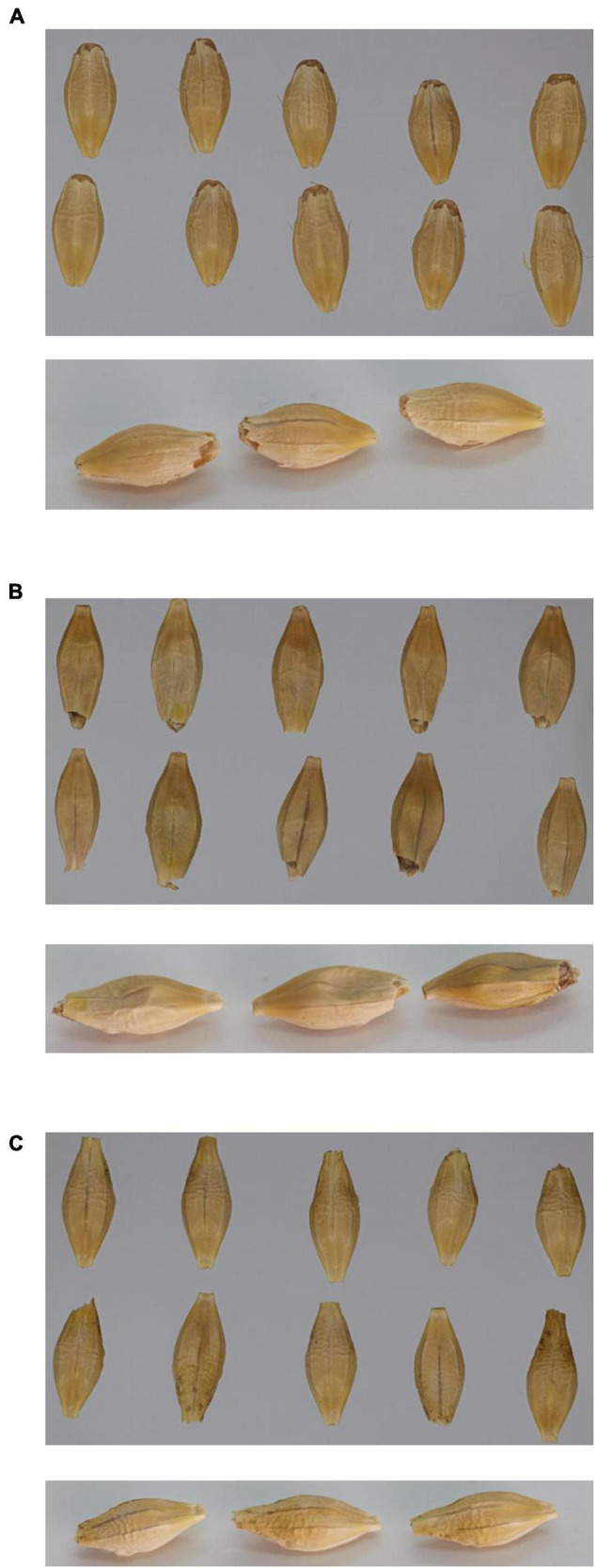
Pictures of 10 mature seeds from above (top picture) and three mature seeds from downward angle (bottom picture). **(A)** Golden Promise; **(B)** GP*^*pdi*^*; **(C)** GP*^*PDI*^*.

Visually, the grain from GP*^*pdi*^* with the lowest 1,000-grain weight had a shrunken grain phenotype compared to wild type GP and GP*^*PDI*^* (Figure 2).

### Microscopy and Protein Staining of GP^*pdi*^ Grains

**FIGURE 3:**
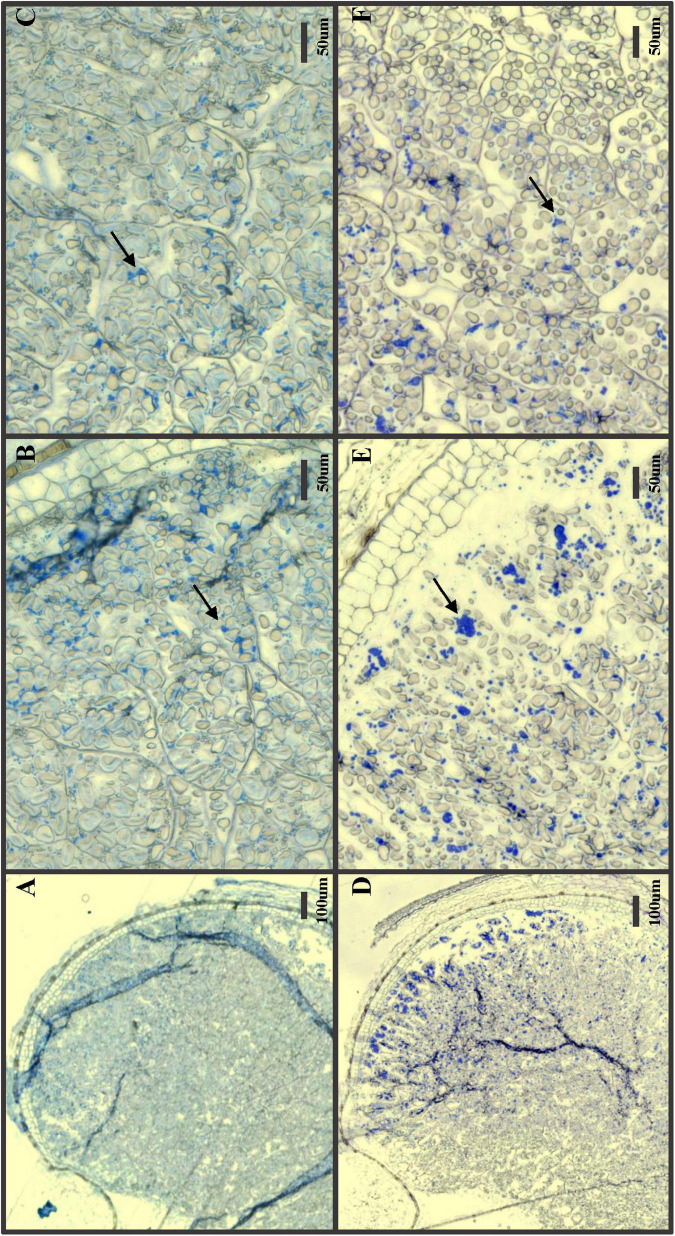
Protein stain of developing seeds 20 days after pollination. **(A–C)** GP*^*pdi*^*; **(D–F)** Golden Promise. **(A,D)** Zoomed out picture with the endosperm and aleurone layer. **(B,E)** Zoomed view of outer are including the aleurone layer. **(C,F)** Zoomed view of inner part of endosperm. Arrows: Protein bodies. Size bars with length indicated in μm.

GP*^*pdi*^* grains with a strongly reduced 1,000-grain weight and shrunken grains were selected for microscopy and protein staining (Figure 3). In GP wild type control, distinct, dark blue protein bodies were clearly visible among starch granules across the entire endosperm, with a denser coloration in the outer cell layers (Figures 3D–F). The protein distribution in GP*^*pdi*^* was considerably less distinct with fewer protein bodies (Figures 3A–C). The endosperm of GP*^*pdi*^* displayed a light blue coloration, almost covering the entirety of the cells. The proteins in the starchy endosperm were not properly assembled into protein bodies, but instead diffused within the cells. From these images, we were not able to determine if there was a reduction of protein within the grain of GP*^*pdi*^* or if it was only the distribution of protein that were affected. We therefore estimated the protein content in mature grain of GP*^*pdi*^*, GP*^*PDI*^*, and GP grown in the semi field. Both samples from GP*^*pdi*^* and GP*^*PDI*^* had a higher protein content than GP wild type with 14, 15.7, and 10.9%, respectively (Table 2). The amount of protein per 1,000 grains were calculated to be 5.01, 4.13, and 5.99 g for GP, GP*^*pdi*^*, and GP*^*PDI*^*, respectively. In conclusion, the knockout of PDI both reduces the amount of protein and disrupts the cellular organization of the protein in the grains of these lines.

### EGF^ORF^ and mLIF^ORF^ in Stress Gene Modulated Barley Grains

#### Stress Gene Overexpression Lines

Recombinant EGF*^*ORF*^* and mLIF*^*ORF*^* was introduced into the stress gene modulated barley plants by crossing. All stress gene modulations were represented by at least one barley plant expressing each of the recombinant proteins, except for GP*^*GST*^* and GP*^*HSP*26^* (Supplementary Table 5). EGF*^*ORF*^* and mLIF*^*ORF*^* lines were used as the maternal parent and pollen from the stress gene modulated plant lines were used as pollinators. F_1_ offspring from crosses were screened by PCR amplifying the hygromycin resistance genes in the two T-DNA constructs (Supplementary Figure 4). The F_1_ offspring plants selected for further analysis were: two crosses of EGF*^*ORF*^* x GP*^*IPI*^* (EGF*^*IPI–*1^* and EGF*^*IPI–*3^*), one cross with GP*^*PDI*^* (EGF*^*PDI*^*) and one with GP*^*HSP*70^* (EGF*^*HSP*70^*). Three crossed plants between mLIF*^*ORF*^* and GP*^*IPI*^* were analyzed (mLIF*^*IPI–*1^*, mLIF*^*IPI–*2^*, and mLIF*^*IPI–*3^*).

#### Stress Gene Knockout Lines

Initially, crossings with knockout mutants were screened for the EGF*^*ORF*^* or mLIF*^*ORF*^* T-DNA constructs (Supplementary Figure 5). Following that, EGF*^*ORF*^* or mLIF*^*ORF*^* positive plants were analyzed for stress gene knockout mutations by PCR/RE (Supplementary Figures 6, 7) and sequencing or by sequencing alone. All plants were F_1_ generation, meaning they are heterozygous for both the recombinant protein construct and the gene mutation. The exception was a cross between GP*^*gst*^* and mLIF*^*ORF*^* which was analyzed in the F_2_ generation. A homozygous mutant (mLIF*^*gst*^*) could be achieved through segregation and selection of offspring from the F_1_ cross (Supplementary Figure 6). A sister plant was identified with a complete PCR digestion, showing an out-segregation of the *GST* mutation, making it a wild type of control (mLIF*^*wt*^*). The remaining F_1_ crossed plants with the other modulated genes were selected for further analysis if they had a heterozygous mutation verified by PCR and sequencing (Supplementary Figure 7). The mLIF*^*ORF*^* was crossed with mutant lines and the selected F_1_ crosses further analyzed were as follows: two crosses with GP*^*crt*^* (mLIF*^*crt–*1^* and mLIF*^*crt–*2^*) and two crosses with GP*^*ipi*^* (mLIF*^*ipi–*1^* and mLIF*^*ipi–*2^*). Offspring from crosses with EGF*^*ORF*^* selected for further analysis were as follows: one cross with GP*^*hsp*70^* (EGF*^*hsp*70^*), one plant from a cross with GP*^*pdi*^* (EGF*^*pdi*^*), and two crosses with GP*^*hsp*26^* (EGF*^*hsp*26–2^* and EGF*^*hsp*26–5^*). A cross with GP*^*hsp*16.9^* was also sequenced without PCR/RE screen (Supplementary Table 5).

### Mouse Leukemia Inhibitory Factor and Epidermal Growth Factor Accumulation in Grains of Stress Gene Modulated Barley

**FIGURE 4:**
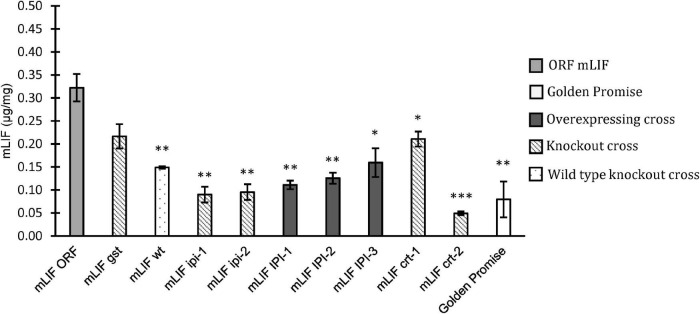
Mouse leukemia inhibitory factor (mLIF) concentration based on ELISA assay in mature grain flour. Golden Promise (white) was used as a negative control. ORF mLIF was used as positive control (light gray). Crossed plants with overexpression (dark gray), crossed plants with knockout mutation (diagonal) and crossed plants with no mutation (dotted). Plants were grown in growth chamber or greenhouse. Error bars represents the standard error and significant differences by Student’s *t*-test. ^∗^*p* < 0.05, ^∗∗^*p* < 0.01 and ^∗∗∗^*p* < 0.001.

The crossed offspring selected above was analyzed for their content of either EGF or mLIF by a sandwich ELISA assay. The mLIF screening was made on plants grown in either a growth chamber or in the greenhouse. The F_2_ plants, mLIF*^*gst*^*, and mLIF*^*wt*^* were grown in a growth chamber. The mLIF concentration in mLIF*^*wt*^* was significantly lower than that in mLIF*^*ORF*^*. The mLIF concentration in mLIF*^*gst*^* was higher than mLIF*^*wt*^* but not to a significant level. The other crosses expressing mLIF were grown in the greenhouse, and all showed a significant reduction on mLIF concentration compared to mLIF*^*ORF*^* (Figure 4). This was the case with both overexpression and knockout of *IPI* and knockout of *CRT* genes.

**FIGURE 5:**
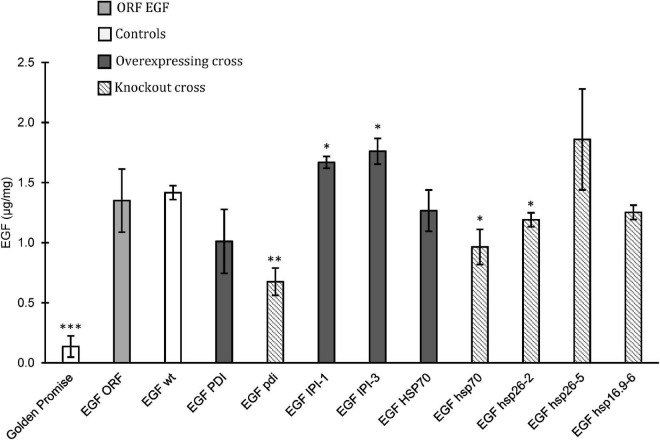
Epidermal growth factor (EGF) concentration based on ELISA assay in mature grain flour. Golden Promise was used as a negative control. Controls (white), ORF EGF positive control (light gray) was grown in growth chamber and greenhouse. Crossed plants with overexpression (dark gray) and crossed plants with knockout mutation (diagonal). Error bars represents standard error. Significant changes found by Student’s *t*-test (^∗^*p* < 0.05, ^∗∗^*p* < 0.01, ^∗∗∗^*p* < 0.001).

The accumulation of EGF in mature grains from crosses with EGF*^*ORF*^* was also analyzed (Figure 5). A F_2_ wild type from a cross with the GP*^*hsp*16.9^* knockout mutant was included as a control (EGF*^*wt*^*). The knockout of *PDI* had a significant negative effect on EGF accumulation showing a decrease from 1.4 μg/mg in EGF*^*wt*^* to.7 μg/mg in EGF*^*pdi*^* (Figure 5). The EGF*^*PDI*^* did, however, not show a positive effect on the accumulation of EGF. The two plants overexpressing *IPI* both had significantly higher EGF content than EGF*^*wt*^* with 1.7 and 1.8 μg/mg for EGF*^*IPI–*1^* and EGF*^*IPI–*3^*, respectively. Overexpression of the *HSP70* gene had no effect on the accumulation of EGF in the offspring whereas EGF*^*hsp*70^* knockout significantly decreased the EGF level. The studied offspring from crossing with GP*^*hsp*26^* showed varying EGF concentration. EGF*^*hsp*26–2^* had a significantly less EGF than the EGF*^*wt*^* control, while the EGF*^*hsp*26–5^* had an increase in EGF to 1.9 μg/mg (Figure 5). However, due to high standard error in the EGF*^*hsp*26–5^* the increased EGF was not significantly higher according to Student’s *t*-test.

## Discussion

The current study has pursued two main objectives. First, to validate the correlation between upregulation of gene expression and protein synthesis during induced ER-stress, as previously described (Barba-Espin et al., 2014). This was done by generating both knockout and overexpression barley lines of seven candidate genes. Secondly, to test if ER-stress can be modulated and facilitate higher capacity for recombinant protein synthesis in grains as demonstrated here with human EGF or mLIF recombinant protein.

We were able to generate knockout mutants for all seven selected genes and overexpressing transformants of five genes. We could not obtain any transformed plants overexpressing CRT or HSP16.9. The reason for this is unknown as the transformations was performed simultaneously with transformations of the other constructs that worked fine. The effect of CRT and HSP16.9 on transformation efficiency will have to be studied further. The ER-stress assay on the obtained lines showed that both knockout mutants and overexpressing transformants had an effect on expression of UPR genes induced by ER-stress. The high expression obtained already at 25 μg/ml TN for many of the knockout and overexpression lines could indicate that the upregulation of UPR genes happened faster than in the GP control. However, at higher TN concentrations, GP increased UPR gene expressions to similar levels as the transformants. This results in the expression of the transformants dropping closer to the baseline with 100 μg/ml TN. A problem with the setup was the uncertainty of the cause of altered UPR gene expression. On the one hand, an increased expression could mean that the cells were prepared for any subsequent ER-stress induction, making them more efficient in alleviating the stress. On the other hand, the increased expression could be due to the cells already being stressed by the overexpression or knockout of the different genes, resulting in the cells being more prone to further ER-stress. Because the candidate genes selected were based on their overexpression during induced ER-stress, it was most likely that overexpressing a gene would have a positive effect in resolving ER-stress, while the knockout of a gene would have a negative effect. The results from the ER-stress assay did show that all candidate genes had some impact on the UPR genes expression, with significant alteration in expression compared to the GP control in at least one of the knockout or overexpressing lines. The change in UPR genes expression was predominantly increased and in correlation with studies on UPR genes expression during ER-stress (Zhao et al., 2007; Lu and Christopher, 2008; McCormack et al., 2015; Uzilday et al., 2018).

The knockout of PDI clearly had an effect on the development of the grain as it showed a shrunken phenotype. The 1,000-grain weight was also 36% less in GP*^*pdi*^* compared to GP wild type. A dissection and staining of protein in the developing endosperm showed a reduced formation of protein bodies, resulting in a diffused protein distribution. Instead of the protein accumulating in the outer cells of the starchy endosperm as in GP wild type, the protein was evenly distributed in the entire endosperm. A protein content measurement on the mature grain showed that the GP*^*pdi*^* mutant had 3.1% more protein in the grain sample compared to GP. The increased protein content in GP*^*pdi*^* mutant could be explained by a concentration effect because grains of GP*^*pdi*^* mutant were much smaller and shriveled than wild type. Therefore, the protein yield per grain was.9 mg lower in GP*^*pdi*^* than that of wild type. These findings are consistent with a previous study showing that HvPDIL1-1 is associated with seed storage proteins during seed development and the starch granule matrix in later developmental stages (Roustan et al., 2018). The knockout of PDI seems here to affect the correct assembly of protein bodies as previously observed in rice. Here, a floury endosperm was observed when PDI expression was impaired (Han et al., 2012). PDI is also implicated in rice endosperm development with the amount and composition of seed storage proteins (Kim et al., 2012).

The difference of the transgene inserts in mLIF*^*ORF*^* and EGF*^*ORF*^* resulted in a large difference in accumulation of recombinant protein. The two copies within the same T-DNA of the *EGF* gene driven by a B-hordein promoter and the oat globulin seed-specific promoter in EGF*^*ORF*^* resulted in 1.4 μg/mg EGF in the ELISA assay, while the mLIF gene driven by the D-hordein promoter in mLIF*^*ORF*^* only resulted in.3 μg/mg mLIF. The main reason for this is the double copy construct of EGF and the use of a stronger promoter of the B-hordein compared to the D-hordein promoter driving the mLIF gene (Furtado et al., 2009). The random integration of the T-DNA in the barley genome by *Agrobacterium* will also influence the expression of the T-DNA genes depending on the integration site. The different complexities of the two recombinant proteins might also have an effect on the stability of the proteins and the ability of the cell to fold and secrete them in large quantities. This could be one of the reasons why we see a positive effect on non-glycosylated EGF accumulation when overexpressing IPI but see no effect on mLIF accumulation which is a highly glycosylated protein with 7 N-glycosylation sites and 2 O-glycosylated sites predicted. It was unexpected that IPI only had a positive effect on EGF accumulation as IPI is part of the mevalonate pathway needed for polyisoprenoid lipid carrier synthesis, particularly for carrying glycans for N-glycosylation (Jones et al., 2009). This indicates a bottleneck of mLIF synthesis that is not affected by more IPI. On the other hand, this could be sufficient for EGF protein synthesis as this does not take up glycan resources since it is not a glycoprotein. The effect of overexpressing IPI might therefore only influence the folding of native proteins or other biosynthetic pathways and thereby increase the capacity of ER synthesis of other proteins including EGF, resulting in the increased EGF accumulation seen in the EGF*^*IPI–*1^* and EGF*^*IPI–*3^*.

One gene showed interesting results in increased EGF accumulation. The overexpression of IPI in EGF*^*IPI–*1^* and EGF*^*IPI–*3^* resulted in increased EGF in the grains, both being significantly higher than an EGF*^*wt*^* cross. Interestingly the IPI did not affect the accumulation of mLIF, but rather significantly decreased the concentration of mLIF in the grain. This shows that any positive effect on recombinant protein from overexpressing *IPI* is depended on the recombinant protein. The reason for this increase is discussed above. The EGF^hsp26–2^ and EGF^hsp70^ knockout mutants both had a significantly negative effect on the EGF accumulation. Another cross with GP^hsp26^ (EGF^hsp26–5^) did, however, show a high accumulation of EGF, but with a large variation. Because of the large variation in the measured samples, the result was not significant. We could see from the data that the lowest measured data point in EGF^hsp26–5^ is higher than all data points measured in EGF^hsp26–2^. We did not expect to see a higher accumulation of EGF from a knockout of a chaperone that is induced during ER-stress (Barba-Espin et al., 2014), but rather a reduction in recombinant protein accumulation as we see in EGF^hsp26–2^ and EGF^hsp70^. The results from EGF^hsp26–5^ should be further studied to explain the high EGF accumulation found in the cross.

Future studies with additional types of heterologous proteins, combinations of gene modulations with positive effects on recombinant protein accumulation and generating homozygous lines of the F_1_ crosses demonstrated here, will further reveal the full potential of the strategy.

## Data Availability Statement

The original contributions presented in the study are included in the article/Supplementary Material, further inquiries can be directed to the corresponding author.

## Author Contributions

HB-P contributed with conception and design of the research. MP conducted most experiments and IH contributed. JB contributed with plant material and ELISA experiment setup and analytical tools. MP performed the experiments. YZ was responsible for the protein staining and microscopic imaging of immature barley seeds. MP wrote the first draft of the manuscript. HB-P and IH reviewed and edited the manuscript. All authors read and approved the manuscript.

## Conflict of Interest

The authors declare that the research was conducted in the absence of any commercial or financial relationships that could be construed as a potential conflict of interest.

## Publisher’s Note

All claims expressed in this article are solely those of the authors and do not necessarily represent those of their affiliated organizations, or those of the publisher, the editors and the reviewers. Any product that may be evaluated in this article, or claim that may be made by its manufacturer, is not guaranteed or endorsed by the publisher.

## Footnotes

1
https://www.eurofins.com/agro
