# Supplementary material for: CRISPR/Cas9 and Transgene Verification of Gene Involvement in Unfolded Protein Response and Recombinant Protein Production in Barley Grain
Source: Front Plant Sci. 2021 Nov 15;12:755788. doi: 10.3389/fpls.2021.755788 (PMC8634432; doi:10.3389/fpls.2021.755788)
Supplement: Supplementary file 1 [file Table_1.DOCX]

# Supporting Data S1

The gene sequences used for overexpression constructs. It is stated whether the sequences are from the old or new barley genome. If the sequence is the same for both assemblies, the new annotation is used. The target sgRNA site is also included on the sequence with the PAM site. The restriction recognition site is underlined and the cut site is between the two bases in italic and bold.

PAM

sgRNA

Amplification primers

#### Protein Disulfide Isomerase (genomic) (HORVU4Hr1G043910)

AGAAATCTCACCGCTCCCCCAGTTCCGCCATGGCGATCTCCAAGGTCTGGATCTCGCTGCTGCTCGCGCTTGCCGTCGTCCTGTCCGCCCCGGCGGCCAGGGCGGAGGAGGCCGCCGCCGCGGAGGAGGCCGCGGCCCCCGAGGCCGTGCTCACCCTGCACGCCGACAACTTCGACGACGCCATCGCCCAGCACCCCTTCATCCTCGTCGAGTTCTACGCCCCATGGTGAGCCCTCGCCTCTGGTATCCTATCCGTGCGCGTGTGGAGTGTTGGTGCGGATCCGACGGGTTTAGATCGTGGTCGGTTTCGAGGGGTCTCAGATTCGTGAATTACGGACCACGGGGGAGGGATCTGGCTTTGGCGATTGGTGCGTCTGGATCGCTGATTCCTCTCTAGTTTCGGTTGGATTTGAAGTTCCTCGAGGGGTTTAAAGGTTGTTGGGCTGGGATTCCACCGTGTCCAACTTTATACTGTAACTATGTAGTAGTGCAGATTCTGCCTGTTCCGACTTGTTTCAGTACAGTCTCACTTAGTCTGGTCTGAATTTTGTCAAAGTCGTTGCTAATTCGTTAAAATTATTTTTTTAAGGTGTGGACACTGCAAGAGCCTGGCACCGGAGGTAATTCTTCTGCATCCTTGGACCACTTAGTTAGCACTGTGTTATATGCTGAATTTATTGATCTCATTCGTTGAATTTCCACTTGTGTGCTGTTGCAGTATGAGAAGGCGGCCCAGCTGTTGAGCAAGCACGACCCAGCGATTGTTCTTGCTAAGGTTGATGCCAACGATGAGAAGAACAAGCCGCTTGCGGGCAAGTACGAGGTCCAGGGCTTCCCTACCCTTAAGATCTTCAGGAACGGAGGAAAGAGCATTCAGGAATACAAGGGTCCCAGGGAGGCCGAGGGAATTGTTGAATACTTGAAGAAGCAGGTTGGCCCTGCTTCCAAGGAGATCAAGGCACCTGAAGATGCCACTTACCTTGAAGACGGCAAGATCCACATTGTAAACTTCTCATCTTGTCTGTTCTTACTTTCATATAGCAATTGCCATATAGCCATGGTTGATGTTCTATGTCTGCTGACTAAGTAATGGTGGCAATTGTATAGGTTGGTGTTTTCACGGAATTCAGTGGCCCTGAGTTTACGAACTTCCTTGAGGTTGCTGAGAAGCTGCGGTCTGATTATGACTTTGGCCACACTGTGCATGCCAACCATCTCCCACGTGGTGATGCAGCAGTGGAGAGGCCAGTGGTCAGGCTATTCAAGCCATTTGATGAGCTCGTTGTTGATAGCAAGGTTATACATTGCTCCCTCTGTAAACTAGTAATTACTCTCTCTGTTGCATATTAGTTATTGCTGATTTTGTACAATTTAGTGACAACTAATATGGAACCAAGGGAGTGTTTATCTATGTTCTTCTTACTTCATACAGATTACTGATGTGATCTTTTGCAGGATTTTGATGTTTCTGCTTTGGAGAAATTCATTGATGCTAGCAGCACCCCGAAAGTTGTTATTTTTGATAAGAACCCTGACAACCATCCGTACCTCTTGAAATTCTTCCAGAGCAATGCTCCCAAGGTAATGACTGACGCAACTTGCTTCTAGAATGCTTTATCATTTTTCGTAATTGTATGAGTTCTGTATAGATGATTAATTTTTACTTTATATGCCTAGTCAGTTAGGTCTATGCCTCATGTTTGTAACTAATGGGGCATTAATGTTGATCACTATATGTTGTTTTGATGGACAGTTGATTACTACTGTATATTTTAGTTGATTATCAGCCCAACTCTTGGTGCGTACACAAAACTTATTTAATCCAATGATGGTTGAATTTGACATCTTTTTTCCTGAAACATGTTGATATAAATGGTTGATTAGTATAGTCTAGTCACTTTTTGGCCCCAAGTCTTTGAATTGGGCAGCTAATTCCTTCAACACGTTTGTTCATCCTGTACATTGAAGAAATCATCTTGCAATGAATTTTTGAAGTGCATTTGGAGTAGTGCATCTAGTTTTGTTAGGCTTAACCTTTTGGGGTTAGTGTTTCCATTCATGGTTGTCGTACCTGAAAGGTTTATTCAGAGCCATATATGTAATGATTGTTTGAGATGCAGCATGGCATGATTTGGTGTTTTCAGTGCTTTGAGCATAAAAATAGTTCAGTTATATGTTTCTAGAGTGGTGAAGCTGCTTAGTGCATTAGCTTTTTCATTGCGTTTTAGTTATTGGTTACTGATTATTGGTCACTTTTGTTATCTGACATCAAATGGTTGCAGGCCATGCTCTTTTTGAACTTCTCCACT***GG***ACCGTTTGAGTCCTTCAAATCAGCCTACTATGGTGCTGTAGAGGAGTTCAGTGGCAAGGATGTCAAGTTCCTAATTGGTGACATTGAATCGAGCCAAGGGGCTTTCCAGGTTGGTTGCTTGTTTCCCTTTCTATCTTAAGTAGTTATACATACTGGTTTTTTTTTTCCTTGGTGCTAACAGAATTTTTTTTGAAACATAGTACTTTGGGCTGAAAGTCGACCAGGCACCACTTATCCTCATTCAAGACGGTGACTCCAAGAAGTTTTTGAAGGAACATGTTGAGGCTGGCCAAATCGTTGCTTGGTTGAAGGATTACTTTGTAAGTAGCCATTCCTAGTTTTTCTTGGTTGAAGGACGACAGCCATATTTGTTTGATCTGGAGGTAATCTCATTAGTGCTCATTCATATATGTAGGATGGTAAATTGACTCCATTCAGGAAGTCCGAGCCTATTCCTGAGGCGAACAATGAGCCTGTGAAGGTAGTTGTGGCTGACAACGTTCATGACGTGGTCTTCAAATCTGGCAAAAATGGTATTCATCTTTGTTAATCTTTTTTCCTCACTGGATTAGTCTAAATGTATCAAGTGACTGGAAATGTTGTCTTGAATGACTTTGGCAAAGTCATCCTGTAAGATATGTGCTCATCGATTCGCCACTCTATATTGCAGTTCTTATTGAGTTCTATGCGCCCTGGTGCGGACACTGCAAGAAGCTAGCACCCATCTTGGACGAGGCAGCTGCCACTCTTCAAAGCGAAGAGGACGTTGTGATCGCGAAGATGGTAAATATTTGTTGCCCTCCCTCGTTTCACTTCCAGCAACGAAGTAGCACAAGTTATCTTAACCACTGTTTCTCGTCCACAGGACGCCACCGAGAATGACGTGCCGGGCGAGTTTGATGTCCAGGGTTACCCGACCCTGTACTTCGTCACTCCCAGCGGGAAGAAGGTCTCTTATGAGGGTGGCAGGACGGCCGACGAGATCGTTGACTACATCAGGAAGAACAAGGAGACTGCTGGGCAGGCGGCGGCGGCGACCGAGAAGGCGGCGGAACCGGCTGCCACCGAGCCTTTGAAGGATGAGCTCTGAGCAACAGTCTTTCTAGCAGCAGACAGGTAGAGGATGGGGAAACATGTTTTGGCAAGGCAGATTCCAACGCCAGATTTTGCGAGGGGGGTCGAGAGTTGGTTGT

#### Glutathione-S-transferase (genomic) (HORVU4Hr1G057890)

CAATGAGTTCGCTCGCTTTCCCCTGCCGCTCGTCACCACTGACGCCCGCCTCCAATTCCCCTCCCGTCCCGTCGCCGTGTATAAAGATCCGCCGCTCGCGACGAGCTGCTCCCCATCGCCACCTCATCGCCGCCAGGAATTCGAGCTCCCCCC***GG***ACCGTCGCCATGGCCGCCGCAGCCGCTGCCGCACCGGTCATGTGAGCAGAGCAGGCCGAGTCGCGGCGCAGAATCTACTGCGTTTGAGTCTCTATTAAATTATTCGAATCGTTTCTCCCTTGTGTTTTTAACTTGGTGTTCGTTTCTCTATCCAACGTGCGAAGCTCCCCGAAAGAAAATCTGCCCCCTTCGCTGACCTCCACCTCCGAGCCCCCGCCACTCTTCGACGGCACGACCAGGTGAATTTGGACAAGGCATACGAGAGATCAAATTGCATTGCAGATAGTATACTACTACTACCCTGTGGCTTGTCCCTTTTGCATAAATCAAGGGTTAACCTGTTGAAGCTTTTTTCCATGTCTCGTGAAGGTTGTATGTTGCATATCACTGCCCGTACGCGCAGCGCGCTTGGATTGCCAGGAATTACAAGGTAACTGTTGTTCTTTGATTTGCTTATGTAATTTGAACAGAAACCGTACTTTTGCTGCTGCTGTGCAAACAACACTGAATCATTCTTACACCTGAATGAAATCTAATTGTTTTTCTTGTCCAGGGTTTGCAAGACAAGATCAAGATAGTCGCAGTCGATCTTGCAGACAGGCCCGCCTGGTACAAGGAGAAGGTTTACCCACAGAACAAGGTATGGAGCATCTCAACTCTGCCCCACGTGTACGATATCTTAACACCGTTTCAGTTTATCATCTCTGTGATCTCCATCCACACTCACGTTGAACTTATTTTTGGACAGGTGCCTTCACTGGAGCATAATAACCAAGTGAAAGGAGAGAGCTTGGATTTGGTCAAGTACATTGACAGCAATTTCGATGGCCCTGCATTGCTTCCTGATGTAAATATTTTGAACCATCATGATCATAATTCATGGTAACTGGGTAAAGCAATTTCGCAGGGGCTTTAATGCTTCCATTCATAACTCTGCAGGATTCTGCAAAGAAGCAGTTTTCCGAGGAGCTGCTTGTGTATACCGATGAGTTCAATAAAGCACTATACTCATCCATAACCTCCAAGGGAGATGTTGCAGAGGAAACTGGTAAAACAACAGAGCCATACCTGAAACTTGTTTAATCTATAGGCTGCAGTCTGTATTCAGAATTGTAACTTCTTATGCATTACCTTCCATTAGTTGCTGCGCTGGATAAAATAGAAGCCGCCCTGGGAAAGTTTAGTGATGGCCCTTTCTTCCTTGGCCAGTTCAGTTTGGTATGTCATGCCGATCCGGGAATCCTTAAAATTCCATTTCAGAAGAATTGTGTTAACATGTTTGACTTGTTGCAATGTTAATTTCTGCACAGGTGGACATTGCATATGTGCCATTTATCGAAAGGTTTCAGATATTCTTTTCTGGTATAAAAAACTATGATATCACCAAGGACAGACCTAACATTCAGAAATTCATCGAGGTAAGTCTGAATGATCCGGGTTTGACTATGCCACATTGTATTGTTATGTTTAAAGTATATTCTCATTTATCGGCAAATGTTAGTCTAATGCAAATCAATTTCCTTTGCAATTCTTTTGCGGTCGAGAGGCAATTTAATCGATATGTTACAGGAAGTGAACAAGATCGATGCATACACACAAACGAAGCTGGACCCACAATTTCTGCTTCAACACACAAAGAAGCGGCTTGGGGTAACAACATATTCTCTAATCTTTCAGCCTTGGTTCACTTTCAACCCATTTTGCACTTACAAACTTCAACTTCCAGATTGAGTGAAGATATGAC

#### HSP70 (genomic) (HORVU4Hr1G059260)

TGCCAGTTTGCGTTCGATTTGATCGGAAGAGATGGCGAGCAAGGGAGGCAACAAGGGGGAGGGCCCTGCCATCGGCATCGACCTCGGCACCACCTACTCCTGCGTCGGCGTCTGGCAGCATGACCGGGTGGAGATCGTCGCCAACGACCAGGGCAACCGCACCACTCCGTCCTACGTCGCATTCACCGACACCGAGCGGCTCATCGGCGACGCCGCCAAGAACCAGGTCGCCATGAACCCCACCAACACCGTTTTTGGTAAGTCCCACCTACGTGCTTCGATGCAAATACGTGCTCTGCCTTCTGCTCTGTTTTAGGCGCGCACCTCAACAACTGTTTCTGAATTTACGCTTCTGAGCAGTCACTTACCACCCTGCTGTGCCATGCAGATGCCAAGCGGCTCATCGGGCGTCGCTTCTCGGACGCATCCGTGCAGTCGGACATGAAGATGTGGCCGTTCAAGGTGATCCCCGGCGCTGGCGACAAGCCGATGATCGTGGTCACCTACAAGGGGGAGGAGAAGACCTTCTCTGCCGAGGAGATATCCTCCATGGTGCTCACCAAGATGAGGGAGATCGCCGAGGCCTTC***CT***CAGCACAACCATCAACAACGCCGTCGTCACTGTCCCGGCCTACTTCAACGACTCCCAGCGCCAGGCCACCAAGGACGCCGGCGTGATCGCCGGTCTCAACGTCATGCGCATAATCAACGAACCCACCGCCGCGGCCATTGCCTACGGCCTCGACAAGAAGGCCACAAGCACCGGGGAGAAGAACGTGCTCATCTTCGACCTCGGCGGCGGCACCTTCGACGTGTCCATCCTCACCATCGAGGAAGGCATATTCGAGGTCAAGTCCACCGCTGGGGACACCCACCTGGGAGGCGAGGACTTCGACAACCGGATGGTGAACCACTTCGTGCAGGAGTTCAAGCGGAAGAACAAGAAGGACATCAGCGGCAACCCAAGGGCGCTCCGGCGGCTGAGGACGGCGTGCGAGAGGGCCAAGAGGACGCTCTCTTCCACCGCCCAGACCACCATTGAGATCGACTCCCTCTATGAGGGGATCGACTTCTACGCGACCATCACCCGTGCCAGGTTCGAGGAGCTCAACATGGACCTCTTCCGCAAGTGCATGGAGCCCGTGGAGAAGTGCCTTCGGGACGCCAAGATGGACAAGACCCAAGTCCACGAAATCGTGCTCGTCGGAGGCTCCACCCGGATCCCCAAGGTGCAGCAGCTCCTCCAGGACTTCTTCAACGGGAAGGAGCTCTGCAAGAGCATCAACCCCGACGAGGCCGTCGCGTACGGAGCCGCCGTGCAGGCCGCCATCCTCAGCGGCGAGGGCAACCAGAAGGTGCAGGACCTGCTCCTGCTCGACGTGACGCCGCTCTCGCTCGGGCTGGAAACAGCCGGAGGCGTGATGACCACTCTGATCCCGAGGAACACCACCATCCCCACCAAGAAGGAGCAGGTCTTCTCCACTTACTCAGACAACCAGCCCGGCGTGCTGATCCAGGTGTACGAGGGCGAGAGGACGAGGACCAAGGACAACAACCTGCTCGGTAAGTTCGAGCTGTCTGGCATCCCGCCGGCGCCCAGGGGAGTGCCCCAGATCACGGTGACCTTCGACATCGACGCGAACGGCATCCTGAACGTGTCCGCGGAGGACAAGACCACCGGGCAGAAGAACAAGATCACCATCACCAACGACAAGGGGCGGCTGAGCAAGGAGGAAATCGAGCGCATGGTGCAGGAGGCGGAGAAGTACAAGTCGGAGGACGAGCAGGTGCGGCACAAGGTGGAGGCCCGCAACGCGCTGGAGAACTACGCGTACAATATGCGCAACACGGTGCGCGACGAGAAGATCGCGTCCAAGCTCCCCGACGACGACAAGAAGAAGATCGAGGACTCCATCGAGGATGCCATCAAGTGGCTCGACGGCAACCAGCTCGCCGAGGCCGAAGAGTTCGAGGACAAGATGAAGGAGCTGGAGAGCATCTGCAACCCCATCATCTCCAAGATGTACCAAGGTGCCGGCCCGGGCGGCGCAGCCGGCATGGACGAGGACATGCCCAGCGGCGGCGCGGGCGCCGGCGGCGGGAGCGGTGCCGGGCCCAAGATCGAAGAGGTGGACTAAGCGAGT

#### HSP26 (genomic) (HORVU4Hr1G063350)

CTTTCCCCATCCTTCCACCGTCGCCGGGGCATGTACTCCCCTCCGCTCTATAAAAGGGGCGCCGCACCGCTCGCCTCCTCC***CA***CCAGGCACACACTGAAATTCAATTCGACGTGGCACATCGGCTTCCGAATACCAACGATCTCCTAGAGACCATCTCAGTCTCTCTGCTTTCACATCTCGATCGGCTTCCCATAGCTCTATCTGGTGCAATGGCTGCAGCGACCGCCCCCTTCGCTCTCGTCAGCCGCCTCTCCCCGGCCGCGCGCCTGCCCATCCGTGCCTGGAGGGCCGCGAGGCCAGCGCCGCTCTGGACCGGGAGAACCCGCCCGCTCTCCGTGGCCTCGGCGGCGCAGGAGAACACGGACAGCTCCGTCGACGTCCAAGTCAGCCAGGACCGGAACGCCGGCAACCAGCAGGGCAATGCGGTCCAGCGCCGCCCTCGTCGCGCTGCTGGATTTGACATCTCCCCGTTCGGTAAGTCCTCTGCTCCACAGTGTCTTTTAACCAGAGGAATTCGTTCCGGCATGGGAGAAGCAGCGCCCGACGTTCGTGGTTTATTTACGGTAACGTATGCAGGGCTGGTGGACCCGATGTCGCCGATGAGAACGATGAAGCAGATGCTGGACACGATGGACCGGCTGTTCGACGACGCCGTGGGGTTCCCGACGGCGCGGCGCTCGCCGGCGGCGGCGGCGGGCGAGATGCCGCGGATGCCTTGGGACATCATGGAGGACGACAAGGAGGTGAAGATGCGGTTCGACATGCCTGGGCTGTCGCGGGAGGAGGTGAAGGTGATGGTGGAGGACGACGCGCTCGTCATCCGCGGCGAGCACAAGAAGGAGGCCGGGGAAGGCCAGGGCGAGGCAGCCGGAGGCGGCGACGGGTGGTGGAAGGAGCGCAGCGTGAGCTCCTACGACATGCGCCTTGCTCTGCCGGACGAGTGCGACAAGAGCCAGGTGCGCGCCGAGCTCAAGAACGGCGTGCTGCTCGTGTCCGTGCCCAAGAGGGAGACCGAGCGCAAGGTCATCGACGTGCAGGTCCAGTGACGAGGTCGCGTGAGGACTGAACCCTGCATCTGAGG

#### HSP16.9 (cDNA) (MLOC_44536.1)

ACAAACCAAAGCAGCACCGACGATGTCGATCGTGAGGCGTAGCAACGCGTTCGACCCCTTCGCCGACCTCTGGGCTGACCCGTTCGACACCTTCCGCTCCATCGTCCCGGCGTTCTCTAGC***AA***CAGCGAGACGGCCGCCTTCGCCAATGCTCGCGTCGACTGGAAGGAGACCCCGGAGGCGCACGTCTTCAAGGCCGACCTTCCCGGCGTGAAGAAGGAGGAGGTCAAGGTGGAGGTGGAGGACGGCAACGTGCTCGTTGTCAGCGGCGAGCGCACTAAGGAGGAGGAGGACAAGAACGACAAGTGGCACCGTGTGGAGCGTAGCAGCGGCAAGTTCGTGCGGCGCTTCCGCTTGCCGGAGGACGCCAAGGTGGAGGAGGTGAAGGCCGGCCTGGAGAACGGTGTGCTCACTGTCACCGTGCCCAAGGCCGAGGTCAAGAAGCCTGAGGTGAAGGCCATCGAGATCTCCGGCTGAATGCATG

#### Isopentenyl diphosphate isomerase (cDNA) (AK250098.1)

ATGGCCGGCACGGGCGACGACGCCGGGATGGACGAGGTCCAGAGGCGCCTCATGTTTGACGACGAATGCATTTTGGTAGATGAACAGGACAACGTTGTTGGTCATGAATCAAAATATACCTGCCATCTGATGGAGAAGATTGAATCTCTGAACCTGCTCCACAGGGCTTTCAGTGTATTCCTTTTCAACTCAAAACACGAGCTGCTACTTCAGCAAAGATCTGCAACAAAGGTTACCTTTCCTTTAGTATGGACCAACACCTGCTGCAGTCATCCTCTGTACCGTGAATCTGAGCTTATTCAGGAAAATTTTCTTGGTGTCAGAAATGCTGCTCAGAGGAAGCTCCTCGATGAGCTGGGCATCCCAGCTGAAGATGTGCCC***GT***CGACCAGTTCACCCCTCTCGGTCGGATGCTTTACAAGGCACCATCTGATGGGAAATGGGGCGAACACGAGTTGGACTACCTGCTGTTCATAGTGCGCGACGTGAAGCTGGTCCCGAACCCGGACGAGGTGGCGGACGTGAAGTACGTGCGCCGGGAGCAGCTGCAAGAGCTCATCCGGCAGGCGGACGCCGGCGAGGGCGGGGTGAAGCTGTCCCCCTGGTTCAGGCTGGTGGTGGACAACTTCCTCATGGGCTGGTGGGAGCACCTGGAGAAGGGCACGCTCGCGGAGGCCGTGGACATGGAGACCATCCACAAGCTCAAGTGA

#### Calreticulin (cDNA) (MLOC_67890.1)

ATGGCGATCCGCCGTGGCTCCTCCTGCGCCGTCCTCGCCCTGCTCGCGCTCGCCTCCGTCGCCGCCGTCGCCGCCGACGTCTTCTTCCAGGAGAAGTTCGAAGATGGCTGGGAAAGCCGGTGGGTCAAGTCTGAGTGGAAGAAGGACGAGAACATGGCTGGTGAATGGAACCACACATCTGGAAAATGGCATGGAGATGCTGAGGACAAAGGTATCCAAACCTCTGAGGACTACAGGTTCTACGCCATCTCTGCGGAGTACCCTGAGTTCAGCAACAAGGACAAGACACTCGTGCTGCAGTTCACGGTGAAGCATGAGCAGAAGCTTGATTGCGGTGGTGGTTACGTCAAGTTGCTTGGAGGTGATGTTGACCAGAAGAAATTCGGTGGCGACACACCCTACGGCATTATGTTTGGACCCGA***TA***TCTGTGGGTACAGCACCAAGAAGGTTCACACTATTCTTACCAAGAACGGCAAGAACCATTTGATCAAGAAGGATGTGCCTTGCGAGACTGATCAGCTGTCGCATGTGTACACTTTGATCATCCGCCCTGATGCTACATACAGCATTCTCATTGACAATGAAGAGAAGCAAACTGGAAGCATCTACGAGCACTGGGATATTCTTCCTCCCAAGGAAATCAAGGACCCAGAAGCTAAGAAGCCAGAGGACTGGGATGACAAGGAGTACATTCCTGATCCTGAGGACGTCAAGCCAGAGGGCTATGATGATATTCCCAAGGAAGTCACTGACCCTGATGCTAAGAAGCCTGAGGATTGGGATGATGAGGAAGATGGTGAATGGACTGCCCCAACCATCCCCAACCCAGAGTACAAGGGCCCATGGAAGCAAAAGAAAATCAAGAACCCTAACTACCAGGGCAAATGGAAGGCACCTATGATTGCCAACCCAGACTTCCAGGATGATCCTTACATCTACGCTTTTGACAGCCTGAAGTACATTGGAATCGAGCTGTGGCAGGTTAAGTCAGGAACGTTGTTTGACAACATTCTCATCACTGACGATGCTGCTTTGGCCAAGACATTTGCCGAAGAGACCTGGGCCAAGCACAAGGATGCTGAGAAGGCTGCTTTTGACGAGGCTGAAAAGAAGAAGGAAGAGGAGGATGCTTCCAAGGCCGGTGAGGACGATGATGACTTGGATGATGAGGATGCCGACGATGAGGACAAGGACGACAAGGCTGGGTCTGATGCTGAGGATGACAAGGATTCTGATGATGAGAAGCACGATGAGCTCTAG
