# Supplementary material for: CRISPR/Cas9 and Transgene Verification of Gene Involvement in Unfolded Protein Response and Recombinant Protein Production in Barley Grain
Source: Front Plant Sci. 2021 Nov 15;12:755788. doi: 10.3389/fpls.2021.755788 (PMC8634432; doi:10.3389/fpls.2021.755788)
Supplement: Supplementary file 2 [file Data_Sheet_1.PDF]

Supplementary Table S1: Target sgRNA sequences including the PAM sequence, either 5-'NGG-3' or complementary 3'-CCN-5' (underlined). Fourth base from the PAM site (red) and the restriction enzyme recognition site in bold.

Gene	sgRNA sequence incl. PAM	Restriction enzyme
HSP70	CCTTC**CTCAG**CACAACCATCAAC	DdeI
HSP16.9	GTTCTCTGGCAACAGC**GAGACG**G	Esp3I
PDI	CCACT**GGACC**GTTTGAGTCCTTC	AvaII
IPI	CC**GTCGAC**CAGTTCACCCCTCTC	SalI
CRT	CC**GATATC**TGTGGGTACAGCACC	EcoRV
GST	CCC**GGACC**GTCGCCATGGCCGCC	AvaII
HSP26	CCA**CCAGG**CACACACTGAAATTC	BptI

Supplementary Table S2 Primer list of primers used to amplify the region of CRISPR/Cas9 mutation. The annealing temperature used in the PCR program, size of PCR product and digested fragments.

Gene	fw (5'-3')	rv (5'-3')	Anneal temp. (°C)	PCR size	digest fragments (bp)
HSP70	CTGCCTTCTGCTCTGTTT	CCTTCTTGTTCTTCCGCTT	58	651	58/236/357
HSP16.9	TTTCCCCGAGCTACAAACCA	CCAATGAATCTTTGCGGCTAC	60	851	134/717
IPI	TGTGGTCGGTCTTTTGGT	TGGGGGTGAGAATGAGGT	58	1415	414/139/862
CRT	CAGCAACAAGGACAAGACA	CTCCTTGTCATCCCAGTC	55*	575	233/342
GST	AACGTACCGGAAGAGCAA	AACACAAGGGAGAAACGA	56	410	283/127
HSP26	CCTCAACCACTACAACGCTACTC	TGCAGCCATTGCACCAGA	61	693	559/134
PDI	AAGTCTTTGAATTGGGCAGCTA	GGAAGTGAAACGAGGGAGGG	59	1219	401/818

*Amplified using touchdown PCR

Supplementary Table S3 Primers used to amplify the gene sequences for overexpression. All primer have a CAGGCTGAGGTCTTAAT 17 nt extension with 15 nt homologous to the vector insertion site and 2 nt (AT) PacI restriction site overhang. The targets were amplified from cDNA (CDS) or genomic DNA (gDNA). The size of the insertions in bp and the number of exons in the gDNA sequence is also noted.

Gene	Primer forward (5'-3')	Primer reverse (5'-3')	Amplified	Size (bp)	Exons
IPI	ATGGCCGGCACGGGCG	TCACTTGAGCTTGTGGATGGTCTCCATG	CDS	708	-
CRT	ATGGCGATCCGCCGTG	CTAGAGCTCATCGTGCTTCTC	CDS	1260	-
HSP16.9	ACAAACCAAAGCAGCACC	CATGCATTCAGCCGGAGA	CDS	453	-
HSP70	TGCCAGTTTGCGTTCGATTT	ACTCGCTTAGTCCACCTCTT	gDNA	2105	2
HSP26	CTTTCCCCATCCTTCCAC	CCTCAGATGCAGGGTTCA	CDS/gDNA	980/1017	2
GST	CAATGAGTTCGCTCGC	GTCATATCTTCACTCAATCTGGAAGTTGAAGTTTGTAAG	gDNA	1896	9
PDI	AGAAATCTCACCGCTCCC	ACAACCAACTCTCGACCC	gDNA	3504	10

Supplementary Table S4: Primer sets used for qPCR analysis in the ER-stress assay and for screening mutant plants for overexpression of target gene. The *SP2* reference gene was used for overexpression screening of mutants and the *actin* and *GAPDH* were used in the ER-stress assay

qPCR Name	Forward primer (5'-3')	Reverse Primer (5'-3')	Target
ER-Stress			
qPCR_Actin*	CCTCAGTTGAGAAGAGCTACG	TCTGCGCCAATCGTGATC	Actin
qPCR_GAPDH*	GCTCAAGGGTATCATGGGTTACG	GCAATTCCAGCCTTAGCATCAAAG	GAPDH
qPCR_PDI	GAAGATGGACGCCACCGAGA	TTCTTCCCGCTGGGAGTGAC	PDI
qPCR_BiP	AGAAAGTTTGAGGACAAGGAGGT	GTACGCAGGAACAGTGACGA	BiP
qPCR_CRT	GCTTGATTGCGGTGGTGGTT	TCTTGGTGCTGTACCCACAGAT	CRT
Overexpressing Mutant Screening			
qPCR_SP2*	GAAGGATGAGTAGGCGCTGG	CTGGGAGGTTCCCAACGTAA	SP2 (splicing factor 2)
qPCR_IPI	GGTAGATGAACAGGACAACG	CTGAAGTAGCAGCTCGTG	IPI
qPCR_GST	ACGGCACGACCAGGTTGTAT	GGCCTGTCTGCAAGATCGAC	GST
qPCR_HSP26	CAGCGTGAGCTCCTACGACA	ACCTGCACGTCGATGACCTT	HSP26
qPCR_HSP70	CGAACGGCATCCTGAACGTG	CTGCTCGTCCTCCGACTTGT	HSP70
qPCR_PDI	GAAGATGGACGCCACCGAGA	TTCTTCCCGCTGGGAGTGAC	PDI
qPCR_CRT	GCTTGATTGCGGTGGTGGTT	TCTTGGTGCTGTACCCACAGAT	CRT

* Reference genes

Supplementary Table S5: Overview of generated overexpression (OE) and knockout mutant (KO) lines and data collected for each. If the mutant line was crossed with ORF EGF or ORF mLIF is also stated.

									Crossings			
**Gene**	**Knockout**	**Overexpression**	**KO**	**OE**	**KO**	**OE**	**KO**	**OE**	**KO**	**OE**	**KO**	**OE**
HSP70	GP^{hsp70}	GP^{HSP70}	yes	yes	yes	yes	-	-	EGF^{hsp70}	EGF^{HSP70}	-	-
HSP16.9	$GP^{hsp16.9}$	-	yes	-	yes	-	-	-	$EGF^{hsp16.9}$	-	-	-
IPI	GP^{ipi}	GP^{IPI}	yes	yes	yes	yes	-	-	-	EGF^{IPI}	$mLIF^{ipi}$	$mLIF^{IPI}$
CRT	GP^{crt}	-	yes	-	yes	-	-	-	-	-	$mLIF^{crt}$	-
GST	GP^{gst}	GP^{GST}	yes	yes	yes	yes	-	-	-	-	$mLIF^{gst}$	-
HSP26	GP^{hsp26}	GP^{HSP26}	yes	yes	yes	yes	-	-	EGF^{hsp26}	-	-	-
PDI	GP^{pdi}	GP^{PDI}	yes	yes	yes	yes	yes	yes	EGF^{pdi}	EGF^{PDI}	-	$mLIF^{PDI}$

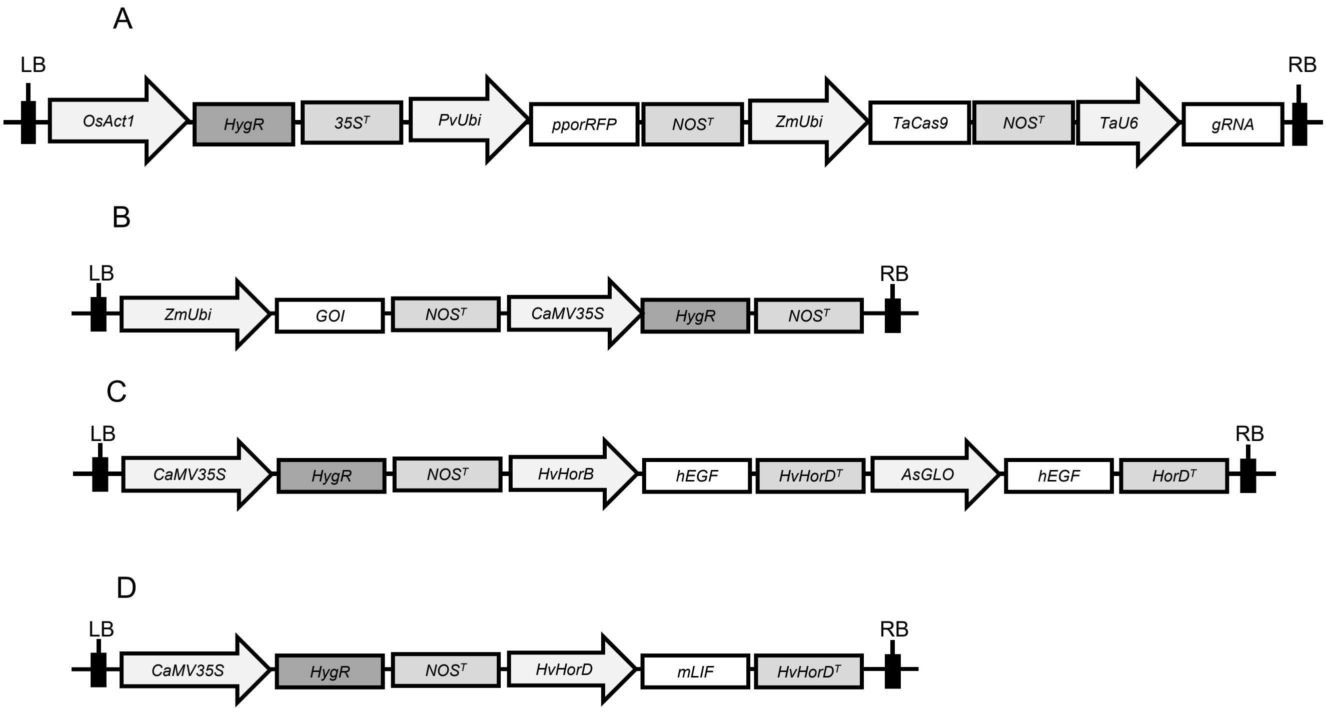

Supplementary Figure S1 T-DNA from constructs. LB: left border. RB: right border. CaMV35S: 35S promoter from Cauliflower mosaic virus. HygR: Hygromycin resistance gene. NOS^T: Nopaline synthase terminator. HvHorB: Barley B-hordein promoter. hEGF: human epidermal growth factor. $HvHorD^T$: Barley D-hordein terminator. AsGLO: Oat globulin promoter. HvHorD: Barley D-hordein promoter. mLIF: mouse Leukaemia inhibitory factor. OsAct1: rice actin1 promoter. PvUbi: Switchgrass Ubiquitin 1 promoter. pporRFP: red fluorescent reporter protein. ZmUbi: maize Ubiquitin-1 promoter with intron. TaCas9: wheat codon-optimized Cas9. TaU6: wheat U6 RNA polymerase III promoter. (A) CRISPR/Cas9 knockout T-DNA. (B) Overexpression T-DNA. GOI: Gene of interest. (C) EGF^{ORF} T-DNA. (D) $mLIF^{ORF}$ T-DNA.

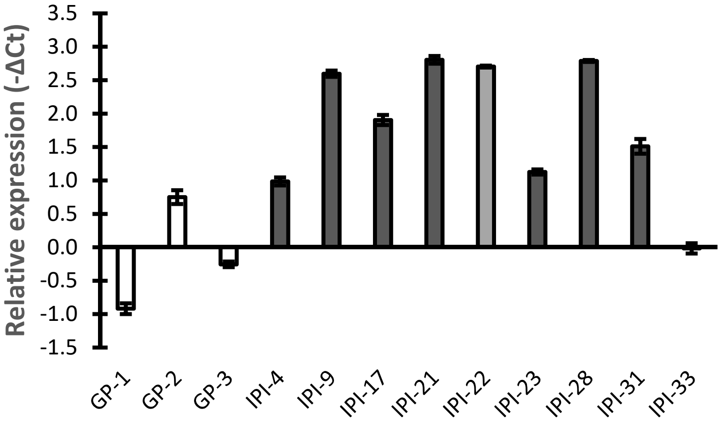

Supplementary Figure S2: Relative expression of target genes in T_0 (IPI, HSP26, PDI and GST) or T_1 (HSP70) mutant plants. GP: Golden promise control (white). Overexpressing transformants in dark grey and selected transformants for further use in light grey. Data represents a technical replicate. Error bars are the standard error

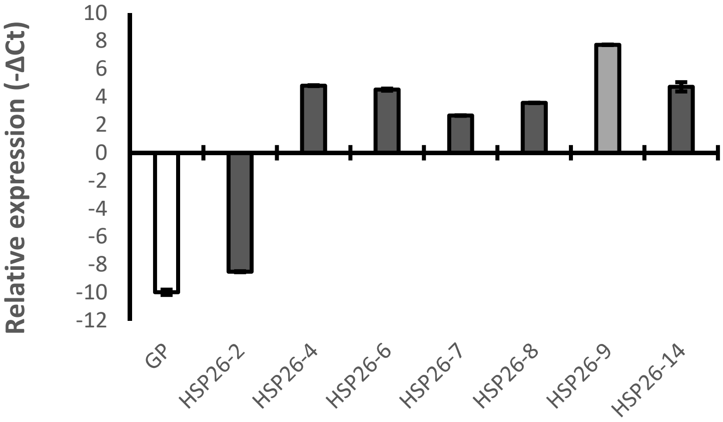

Selected overexpression transformants
Golden Promise
Overexpression transformants

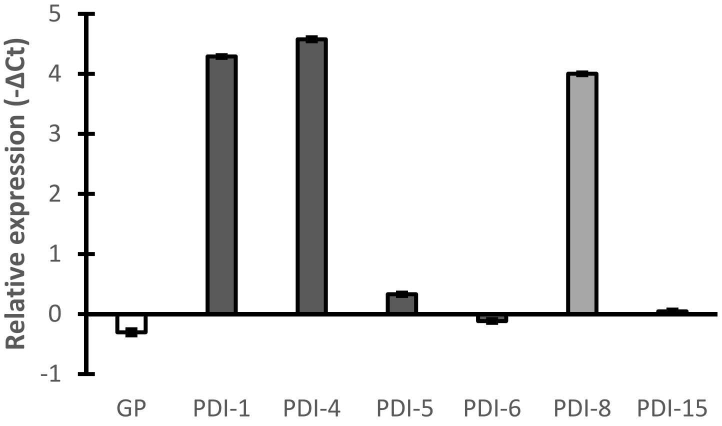

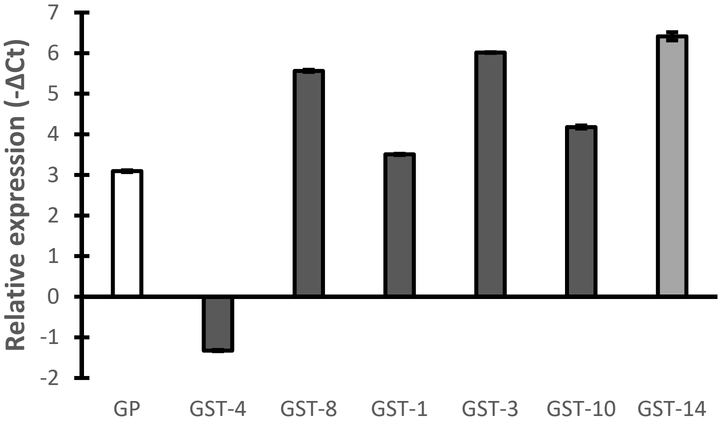

Supplementary Figure S2 continued.

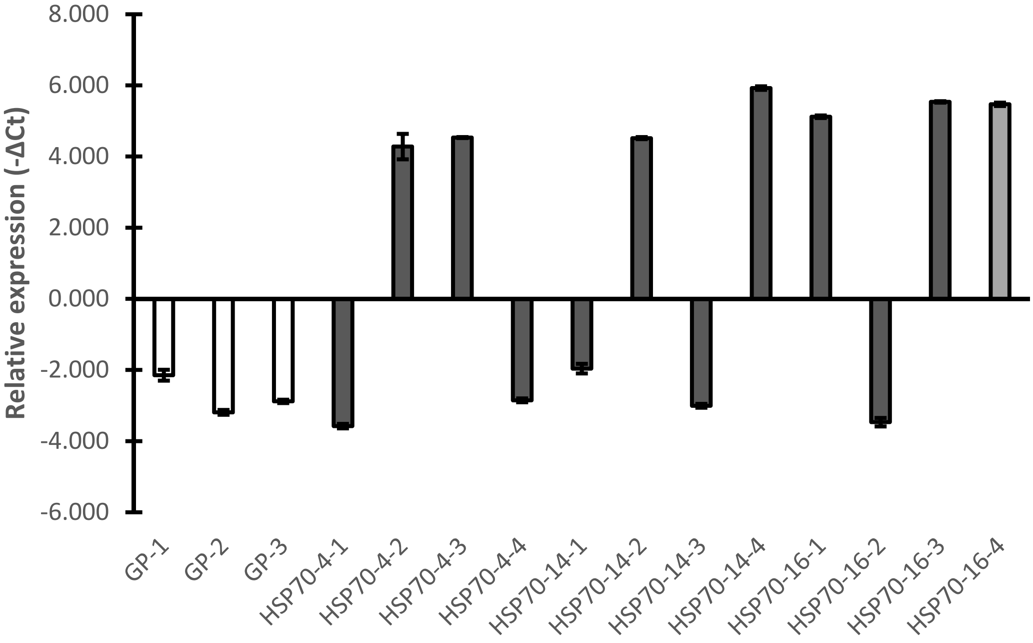

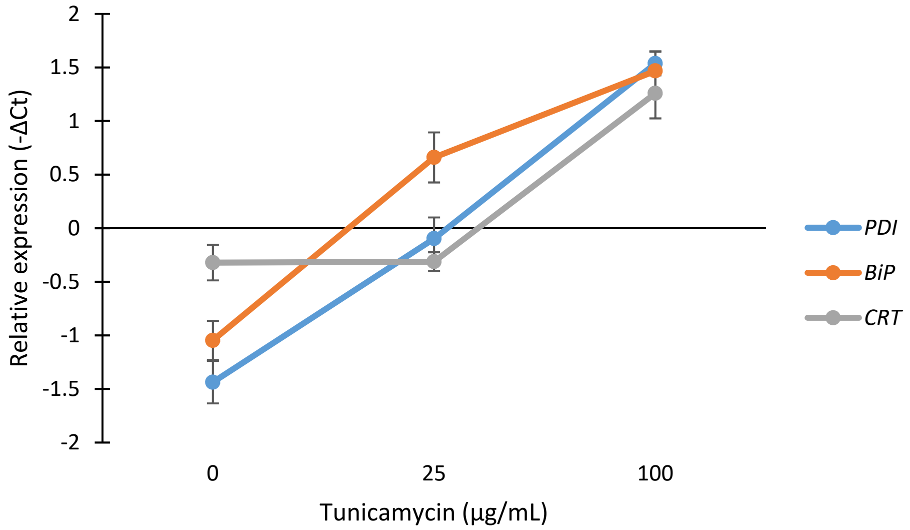

Supplementary Figure S3: Relative expression of UPR genes *PDI*, *BiP* and *CRT* in GP plantlets. The gene expressions were normalized to two reference genes *actin* and *GAPDH.* Expression was measured at three different concentrations of tunicamycin. Data represents a biological triplicate. Error bars are the standard error

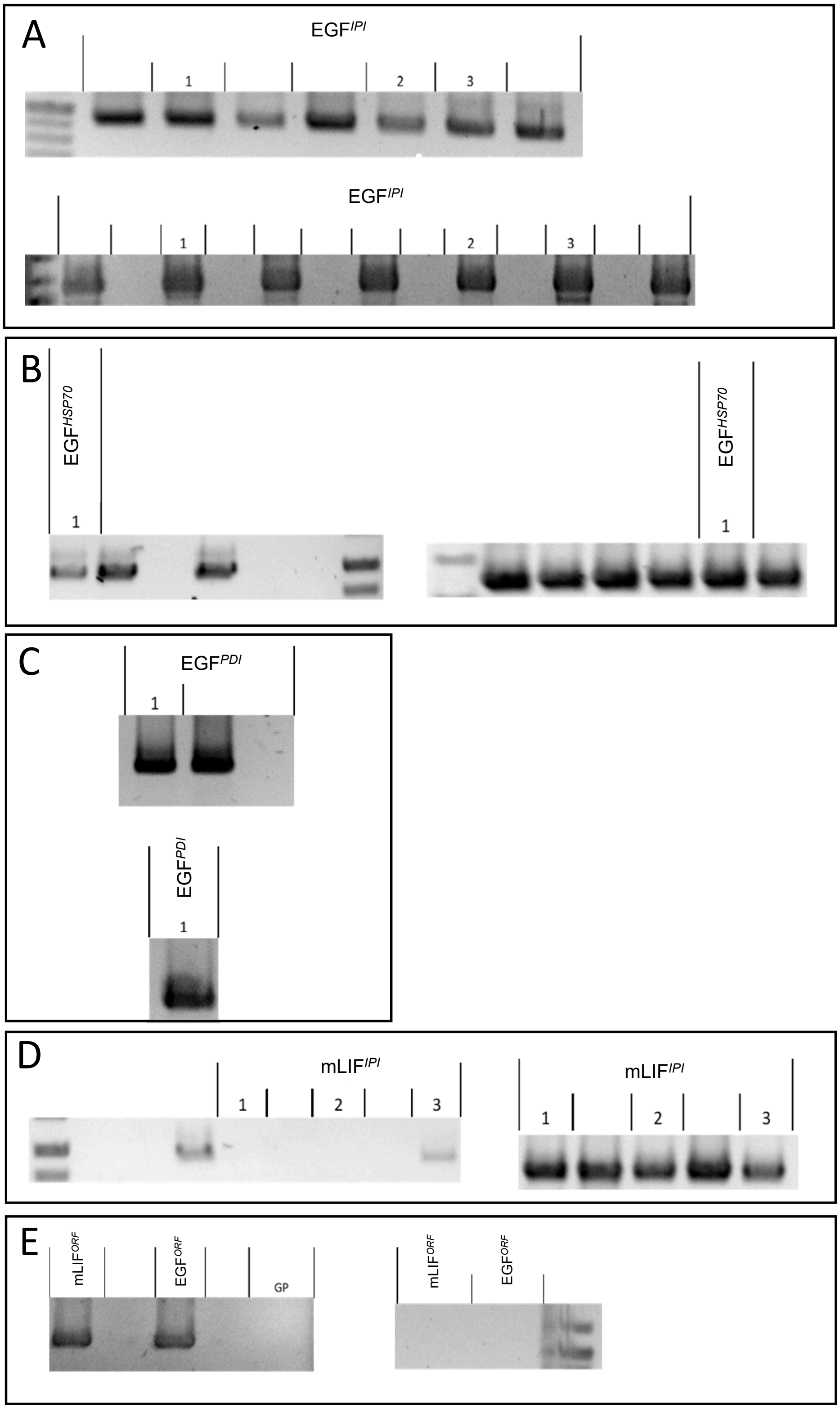

Supplementary Figure S4: PCR screening of the hygromycin resistance genes in crossings with overexpressing lines. First gel is PCR of the gene from the overexpressing parent. The second gel is PCR of the hygromycin gene from the EGF^{ORF} or $mLIF^{ORF}$ parent. A: EGF^{IPI}. B: EGF^{HSP70}. C: EGF^{PDI}. D: $mLIF^{IPI}$. E: EGF^{ORF}, $mLIF^{ORF}$ and Golden Promise (GP) controls.

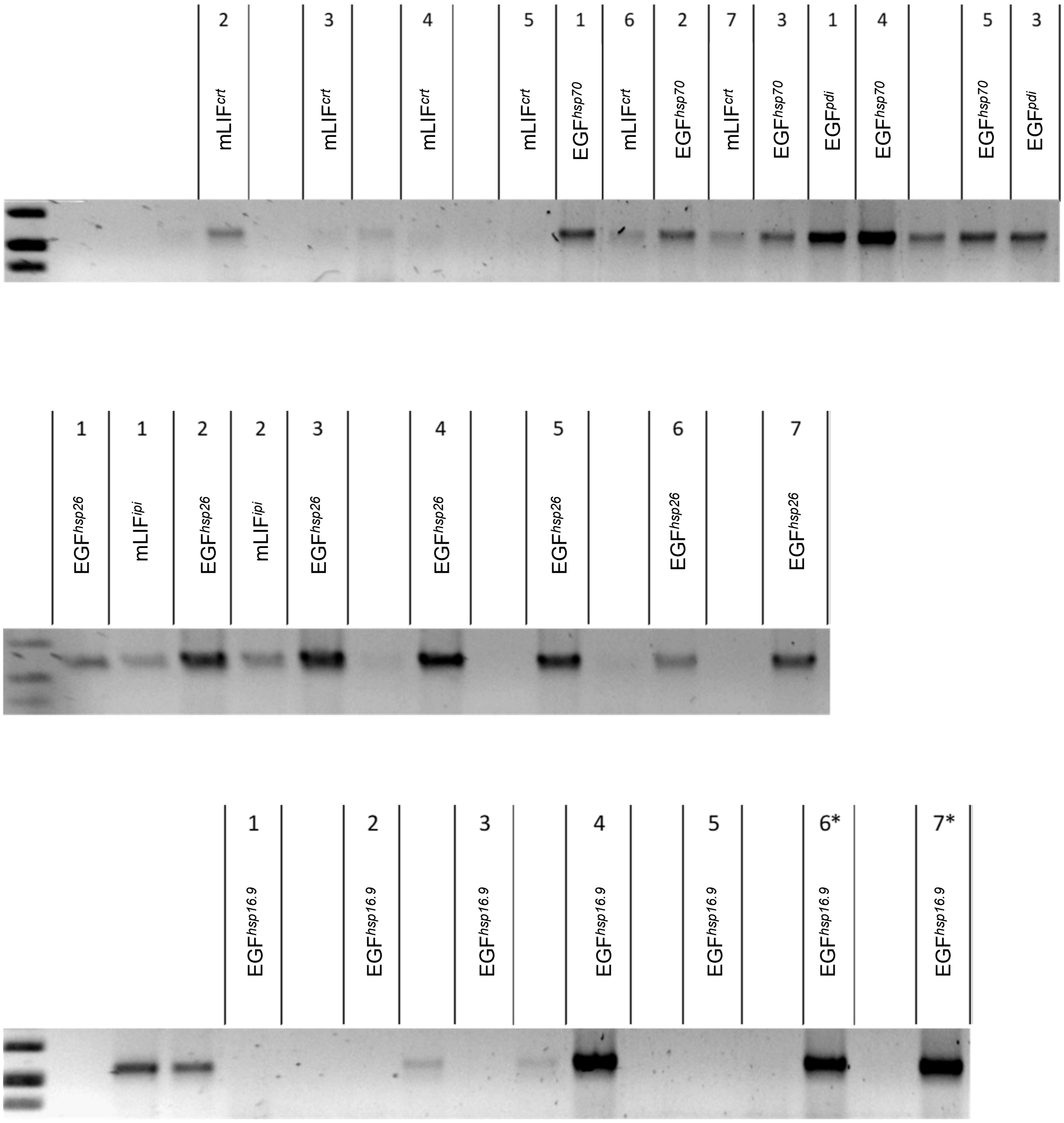

Supplementary Figure S5: PCR screening of F_1 crossings of knockout mutants. PCR amplifying hygromycin resistance gene in the ORF construct. The plant number is stated above. 1000 bp ladder is used with the middle lane = 1000 bp. * = Plants only sequenced for mutation, not PCR/RE.

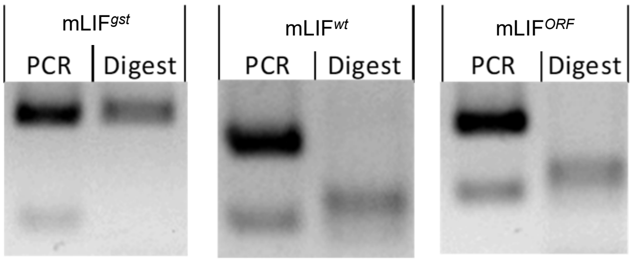

Supplementary Figure S6: PCR/RE screening of F_2 crossings (mLIF^{ORF} X GP^{gst}). One mutant plant (mLIF^{gst}), one wild type plant (mLIF^{wt}) and one control plant (mLIF^{ORF}).

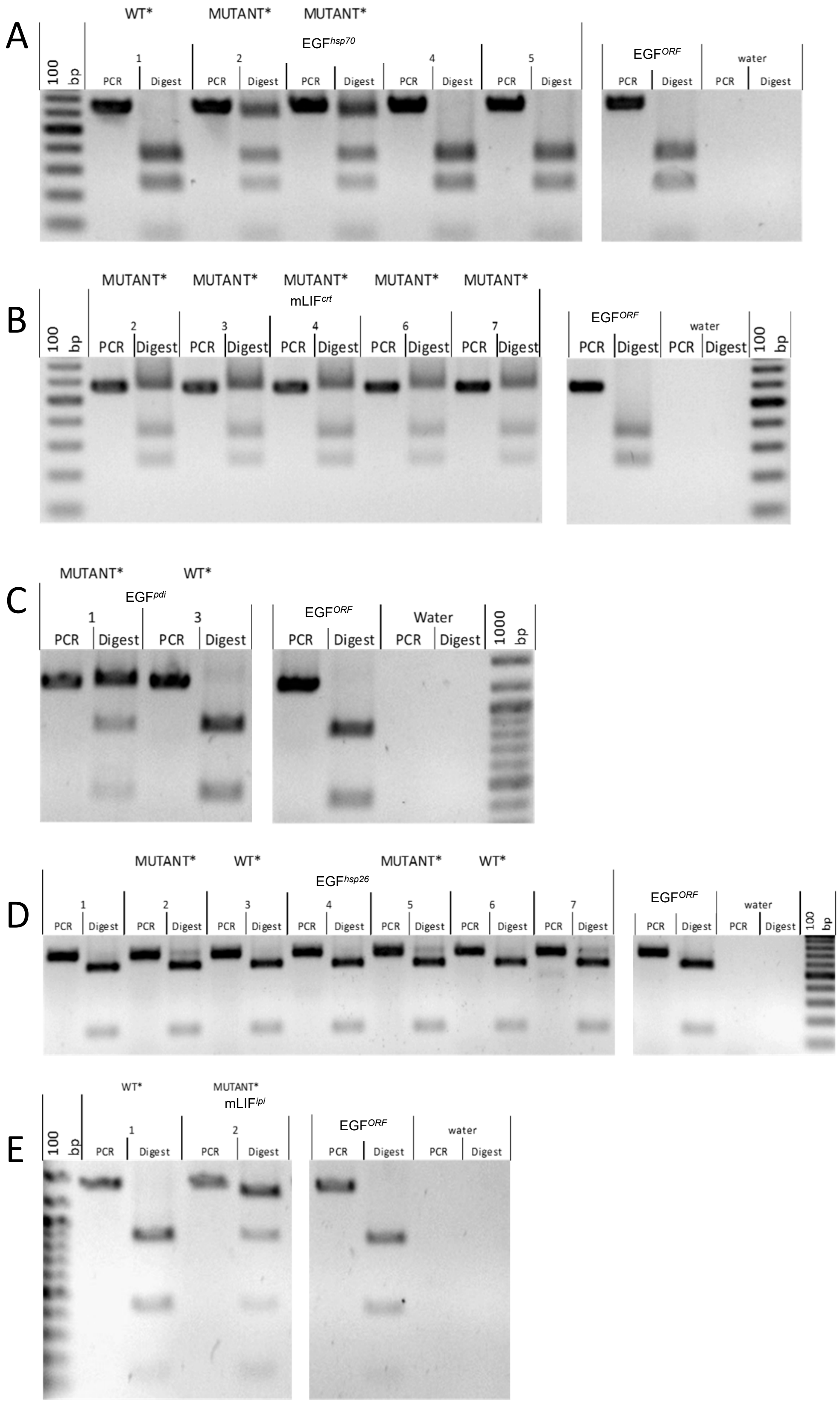

Supplementary Figure S7: PCR/RE screening of crossings with knockout mutants. Undigested (PCR) and digested (digest) of PCR fragments from F_1 crossings. Each number is an individual plant. A: EGF^{hsp70}. B: mLIF^{crt}. C: EGF^{pdi}. D: EGF^{hsp26}. E: mLIF^{ipi}. 100 or 1000 bp ladder stated. Selected plants were sequenced as wild type (WT*) or heterozygous mutants (MUTANT*).
